# Pre-mRNA Processing Factors and Retinitis Pigmentosa: RNA Splicing and Beyond

**DOI:** 10.3389/fcell.2021.700276

**Published:** 2021-07-28

**Authors:** Chunbo Yang, Maria Georgiou, Robert Atkinson, Joseph Collin, Jumana Al-Aama, Sushma Nagaraja-Grellscheid, Colin Johnson, Robin Ali, Lyle Armstrong, Sina Mozaffari-Jovin, Majlinda Lako

**Affiliations:** ^1^Biosciences Institute, Newcastle University, Newcastle upon Tyne, United Kingdom; ^2^Faculty of Medicine, King Abdulaziz University, Jeddah, Saudi Arabia; ^3^Department of Biological Sciences, Durham University, Durham, United Kingdom; ^4^Leeds Institute of Molecular Medicine, University of Leeds, Leeds, United Kingdom; ^5^King’s College London, London, United Kingdom; ^6^Medical Genetics Research Center, Mashhad University of Medical Sciences, Mashhad, Iran; ^7^Department of Medical Genetics, Faculty of Medicine, Mashhad University of Medical Sciences, Mashhad, Iran; ^8^Department of Cellular Biochemistry, Max Planck Institute for Biophysical Chemistry, Göttingen, Germany

**Keywords:** retinitis pigmentosa, pre-mRNA processing factor, spliceosome, gene therapy, splicing, animal models, circadian rhythm, DNA damage and repair

## Abstract

Retinitis pigmentosa (RP) is the most common inherited retinal disease characterized by progressive degeneration of photoreceptors and/or retinal pigment epithelium that eventually results in blindness. Mutations in pre-mRNA processing factors (*PRPF3, 4, 6, 8, 31, SNRNP200, and RP9*) have been linked to 15–20% of autosomal dominant RP (adRP) cases. Current evidence indicates that *PRPF* mutations cause retinal specific global spliceosome dysregulation, leading to mis-splicing of numerous genes that are involved in a variety of retina-specific functions and/or general biological processes, including phototransduction, retinol metabolism, photoreceptor disk morphogenesis, retinal cell polarity, ciliogenesis, cytoskeleton and tight junction organization, waste disposal, inflammation, and apoptosis. Importantly, additional PRPF functions beyond RNA splicing have been documented recently, suggesting a more complex mechanism underlying *PRPF*-RPs driven disease pathogenesis. The current review focuses on the key RP-*PRPF* genes, depicting the current understanding of their roles in RNA splicing, impact of their mutations on retinal cell’s transcriptome and phenome, discussed in the context of model species including yeast, zebrafish, and mice. Importantly, information on PRPF functions beyond RNA splicing are discussed, aiming at a holistic investigation of *PRPF*-RP pathogenesis. Finally, work performed in human patient-specific lab models and developing gene and cell-based replacement therapies for the treatment of *PRPF*-RPs are thoroughly discussed to allow the reader to get a deeper understanding of the disease mechanisms, which we believe will facilitate the establishment of novel and better therapeutic strategies for *PRPF*-RP patients.

## Introduction

Retinitis pigmentosa (RP) is the most common group of inherited retinal disorders characterized by progressive degeneration of photoreceptors and/or the retinal pigment epithelium (RPE). Most RP cases start with night blindness due to the breakdown of rod photoreceptors, which are responsible for night vision. As the disease progresses, mid-peripheral vision is lost (tunnel vision), followed by cone degeneration leading to central vision loss until eventual blindness (Hartong et al., 2006; Berger et al., 2010). The prevalence of RP is around 1 in 4000 and there are over 1.5 million people suffering from this condition worldwide (Verbakel et al., 2018). There is no cure for RP although vitamins, nutritional supplementation and small molecules may slow disease progression.

To date, more than 70 genetic loci have been involved in the pathogenesis of RP^1^. Approximately half of RP cases have previous family history and fall into three Mendelian modes of inheritance: autosomal recessive (arRP), autosomal dominant (adRP), and X-linked recessive (xlRP) (Hamel, 2006). 50–60% of RP cases are caused by autosomal-recessive inheritance, 30–40% of cases are autosomal dominant, and 5–15% of cases are X-linked. Most of the genes involved in RP ontology are expressed specifically in the retina and/or RPE and contribute to photoreceptor or RPE function. Mutations in the rhodopsin (*RHO*) gene are the most common cause of adRP accounting for 25% of adRP cases. Interestingly, the second most common cause of adRP accounting for 15–20% of adRP cases, is linked to mutations in the ubiquitously expressed pre-mRNA processing factor (*PRPF*) genes, that encode core components of the spliceosome (Wang et al., 2019).

**FIGURE 1:**
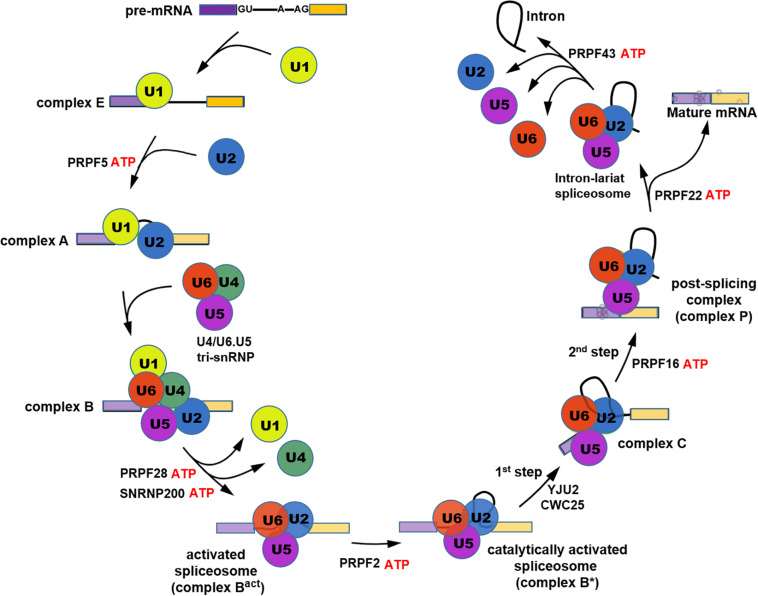
Schematic illustration showing the process of pre-mRNA splicing cycle.

Pre-mRNA splicing is the process which removes introns and ligates exons to form mature mRNA molecules. Splicing occurs in a large RNA-protein complex called spliceosome that is composed of five small nuclear ribonucleoproteins (snRNPs) namely U1, U2, U4, U5, and U6 and approximately 200 additional factors (Wang et al., 2015). The U1 and U2 snRNPs, respectively, bind to pre-mRNA intronic 5′-splice site and branch point site to form complex A followed by recruitment of the U4/U6.U5 tri-snRNP complex to form the precatalytic spliceosome (complex B). Next U1 and U4 snRNPs are released from the spliceosome by the action of RNA helicases PRPF28 and SNRNP200 forming the activated spliceosome (Bact), which is then converted to the catalytically activated spliceosome (B^∗^) that catalyzes two steps of *trans*-esterification reactions, in a stepwise manner, to complete pre-mRNA splicing (Shi, 2017; Figure 1). PRPFs are essential components of tri-snRNP and are critical for the assembly of mature splicing complex. Mutations in seven *PRPF* genes have been identified in adRP, including *PRPF3*, *4*, *6*, *8*, *31*, *SNRNP200* (*Brr2*), and *RP9* (*PAP-1*) (Mordes et al., 2006; Chen et al., 2014; Daiger et al., 2014; Ruzickova and Stanek, 2017), all of which except *RP9* encode components of tri-snRNP and play important roles in the assembly of spliceosome complex B. During spliceosome assembly, U4 and U6 snRNP strongly bind with each other via snRNA paring to form U4/U6 di-snRNP which subsequently associates with U5 snRNP leading to U4/U6.U5 tri-snRNP conformation (Nguyen et al., 2015). Amongst the seven RP-PRPF proteins, PRPF3, PRPF4, and PRPF31 are U4/U6 snRNP-specific factors, while PRPF6, PRPF8, and SNRNP200 are components of U5 snRNP and RP9 is a non-snRNP splicing factor (Liu et al., 2006; Supplementary Table 1).

**FIGURE 2:**
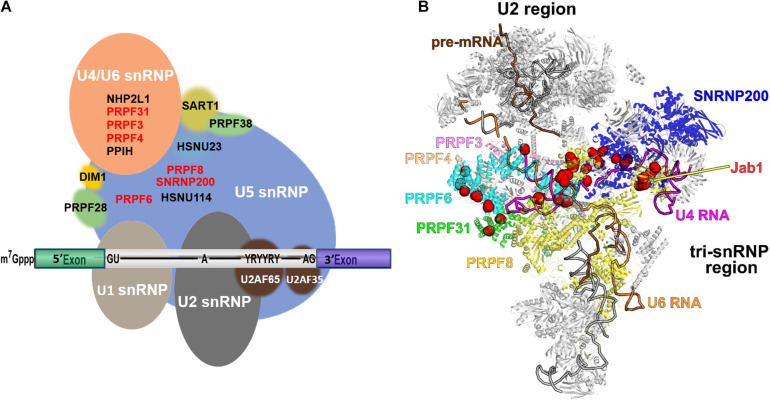
Schematic diagram illustrating the structures of spliceosome complex B and tri-snRNP. **(A)** Composition of complex B; **(B)** cryo-EM structure of pre-catalytic spliceosome complex B with RP-linked PRPFs highlighted. The pre-mRNA, U4, and U6 snRNAs are colored brown, magenta, and orange, respectively. Six PRPFs, which are core components of tri-snRNP, are labelled and color-coded. Residues mutated in RP are shown as red spheres. The PRPF8 Jab1 domain is labelled. The image was created using PyMol (DeLano, 2002).

The interactions among PRPFs and PRPF-snRNA crosslinks within tri-snRNP have been well described in other reviews (Abovich et al., 1990; Makarov et al., 2000; Gonzalez-Santos et al., 2002; Kuhn et al., 2002; Makarova et al., 2002; Nottrott et al., 2002; Schaffert et al., 2004; Liu et al., 2006). Moreover, recent yeast and human cryo-EM structures of spliceosomes at different stages of splicing have shed light on the RNA and protein structural rearrangements of the spliceosome during splicing cycle. Figure 2 shows a snapshot of the human spliceosome complex B after the release of U1 snRNP. Hotspots for RP mutations within PRPF structures are depicted with red spheres. Of note, most of these mutations are in domains associated with RNA binding (e.g., in SNRNP200’s U4 snRNA binding channel).

*PRPFs* are ubiquitously expressed and mutations in RP-*PRPF* genes disrupt spliceosome assembly, leading to faulty splicing (Tanackovic et al., 2011b). However, the mutations appear to be tolerated by the majority of human tissues yet cause non-syndromic RP restricted to the eye. Although the molecular mechanisms underlying this phenomenon remain unclear, several models have been proposed to explain the photoreceptor-specific phenotype. Haploinsufficiency, dominant-negative and gain-of-function are all implicated in the pathogenesis of *PRPF*-RPs (Mordes et al., 2006). As one of the most metabolically active tissues, retina has the highest level of steady state snRNAs and processed pre-mRNAs, indicating the requirement of a higher splicing activity compared to other tissues (Tanackovic et al., 2011b). Thus, the disrupted RNA splicing activity due to *PRPF* mutations is more likely to impair retinal cell functions or viability than other tissues. Using a patient-specific cell model, our research revealed that *PRPF31* mutations induce retinal-specific global spliceosome dysregulation, resulting in mis-splicing and/or differential expression of a subset of genes that play essential roles in retina and RPE cells (Buskin et al., 2018). Aside from their roles in RNA splicing, PRPFs have recently been implicated in photoreceptor ciliogenesis, circadian regulation, and DNA damage repair, suggesting a possible model in which *PRPF* mutations impair crucial retinal functions and contribute to RP development. These extra-spliceosome functions of PRPFs will provide a new perspective on the relationship between distinct RP symptoms and mutations in the ubiquitously expressed splicing factors.

In this manuscript, we review *PRPF* mutations responsible for RP pathogenesis and their role in both splicing and other cellular processes. Disease modeling of PRPF-RP in various species is also discussed to provide more details on RP pathogenesis and development of therapies for *PRPF*-RP.

## *PRPF* Mutations Implicated in RP Pathogenesis

Of all the seven splicing factors, *PRPF31* is the most prominent adRP related gene, whose mutations account for 8.9% of all cases, followed by *PRPF8* (2.6%), *SNRNP200* (1.6%), *PRPF3* (1.5%) and *PRPF4*, *PRPF6*, and *RP9*, which are responsible for a small proportion of adRP occurrence (Bowne et al., 2013; Daiger et al., 2014). Homozygous knockout or mutation of RP-*Prpf* genes is embryonic lethal to animal models (Bujakowska et al., 2009; Linder et al., 2014), implying that these splicing factors are essential for early embryonic development and survival. Heterozygous depletion or mutation of RP-*Prpf* genes lead to development of RP phenotype through loss-of-function and/or dominant-negative effects (Supplementary Table 1). Below we review the types of *PRPF* mutations involved in RP.

The *PRPF3* gene is located on chromosome 1q21.2. The encoded PRPF3 protein is an essential component of U4/U6 snRNP that binds U6 snRNA (Liu et al., 2006, 2015). In the spliceosome B complex, PRPF3 interacts with PRPF4 and PRPF6 and stabilizes U4/U6.U5 tri-snRNP (Figure 2). PRPF3 inactivation compromises spliceosome assembly due to the absence of intact U4/U6.U5 tri-snRNPs (Anthony et al., 1997; Heng et al., 1998; Liu et al., 2006). Heterozygous mutations in the *PRPF3* gene lead to RP type 18 (RP18). The implication of *PRPF3* mutation in adRP was first reported by Chakarova et al. (2002). To date, 10 *PRPF3* mutations have been identified in RP18 families (Chakarova et al., 2002; Amsterdam et al., 2004; Comitato et al., 2007; Gamundi et al., 2008; Graziotto et al., 2008, 2011; Zhong et al., 2016). Eight missense *PRPF3* mutations identified in adRP families are clustered at the highly conserved C-terminal domain for binding to U4/U6 snRNA and splicing factors including PRPF4, PRPF6, p110, SPF30, Hsnu66, and RP9 (Chakarova et al., 2002; Maita et al., 2005; Gamundi et al., 2008; Cao et al., 2011; Zhong et al., 2016). Thr494Met is the most common mutation that is observed frequently in unlinked families worldwide. Thr494Met mutation substantially reduces the phosphorylation of PRPF3 C-terminal region by casein kinase II, thereby suppressing its association with itself, Hprp4p, and U4/U6 snRNA (Gonzalez-Santos et al., 2008). RP18 patient lymphoblast cell lines with mutated *PRPF3* showed altered levels of snRNPs, delayed spliceosome assembly and decreased splicing efficiency (Tanackovic et al., 2011b).

The *PRPF4* gene is located on chromosome 9q32, which encodes a protein associated with RP type 70 (RP70) (Heng et al., 1998). At the entire C-terminal portion of PRPF4 protein, there are 7 WD40 domain repeats that act as protein interaction scaffolds in multiprotein complexes. PRPF4 protein interacts directly with PRPF3 in U4/U6 and U4/U6.U5 snRNPs to play important roles in spliceosome assembly (Anthony et al., 1997; Wang et al., 1997). The link between *PRPF4* mutations and RP was first identified in two labs in 2014 (Chen et al., 2014; Linder et al., 2014). Chen et al. (2014) identified two heterozygous mutations in *PRPF4* in a simplex RP patient and an adRP family. The mutation c.-114_-97del disrupts the binding sites for two transcription factors c-Ets-1 and GR-alpha in ARPE19 and 293T cells and dramatically suppresses the expression of *PRPF4*. Interestingly, in RP patient cells with *PRPF4* c.944C > T (p.Pro315Leu) mutation, tri-snRNP component genes are upregulated, indicating a compensatory response to PRPF4 dysfunction. In addition, both the wild type and mutant *PRPF4* are upregulated, implicating a dominant-negative effect. In 2014, Linder et al. reported another RP associated *PRPF4* mutation c.575G > A (p.Arg192His) that interrupts the interaction between PRPF4 and PRPF3 and incorporation of PRPF4 into tri-snRNP in Hela and Hek293 cells. Loss-of-function rather than dominant-negative effect was suggested as the main mechanism underlying Arg192His mutation related RP (Linder et al., 2014). There are currently 5 PRPF4 mutations identified.

The *PRPF6* gene is located on chromosome 20q13.33 that encodes PRPF6 protein associated with RP type 60 (RP60). PRPF6 protein contains tetratrico peptide repeats (TPR) domains in the C-terminal region that mediates protein-protein interactions and the formation of multiprotein complexes. As a U5 specific protein, PRPF6 serves as a scaffold bridging U5 and U4/U6 snRNPs (Figure 1) during the assembly of tri-snRNPs (Makarov et al., 2000). With *PRPF6* mutation, tri-snRNP accumulation is suppressed (Galisson and Legrain, 1993). Tanackovic et al. (2011a) first identified a *PRPF6* missense mutation, c.2185C > T (p.Arg729Trp) that was co-segregated with adRP in an affected family. PRPF6 mutant protein displays abnormal localization in Cajal bodies within nuclei in Hela cells, where immature snRNP complexes accumulate, indicating an impairment in tri-snRNP assembly or recycling (Schaffert et al., 2004). In addition, RP patient lymphoblasts containing p.Arg729Trp mutation in PRPF6 displayed defective pre-mRNA splicing capacity with signature introns retained, indicating an inefficient spliceosome (Tanackovic et al., 2011a). 18 *PRPF6* mutations have been identified till now.

The *PRPF31* gene is located on chromosome 19q13.42. The encoded pre-mRNA processing factor is linked to adRP type 11 (RP11). PRPF31 is a U4/U6 specific protein that binds directly to U4 snRNA (Figure 1; Nottrott et al., 2002). PRPF31 interacts with PRPF6 and tethers U5 snRNP to the U4/U6 snRNP facilitating the formation of tri-snRNPs (Makarova et al., 2002). PRPF31 contains a Nop domain that mediates protein-RNA interactions (Liu et al., 2007). The knockdown of *PRPF31* by RNAi results in the interruption of U5 and U4/U6 snRNP interaction and inhibition of tri-snRNP formation, demonstrating an essential role of PRPF31 in tri-snRNPs assembly. Similar to *PRPF6* depletion, *PRPF31* knockdown in Hela cells, leads to accumulation of U4/U6 di-snRNPs in subnuclear organelle Cajal bodies, but U5 snRNPs largely remain in nucleoplasmic speckles (Schaffert et al., 2004). Deletion of the *PRPF31* gene in yeast inhibits tri-snRNP assembly and blocks pre-mRNA splicing (Weidenhammer et al., 1996, Weidenhammer et al., 1997). The first RP-related *PRPF31* mutation was reported by Vithana et al. (2001). To date, 229 *PRPF31* mutations of various types have been identified in RP11 families and sporadic cases, including missense/nonsense substitution, deletion, insertion, frameshift, premature stop codon and intron splice site mutation etc. (Mordes et al., 2006; Ruzickova and Stanek, 2017)^2^. These mutations result in alteration of sequence and structure of the PRPF31 protein or a decreased level due to impacts on transcription and pre-mature mRNA/protein degradation, leading to functional defects, haploinsufficiency and dominant-negative effects (Vithana et al., 2001; Martinez-Gimeno et al., 2003; Wang et al., 2003; Xia et al., 2004; Wilkie et al., 2008).

The *PRPF8* gene is located on chromosome 17p13.3 associated with RP type 13 (RP13). The encoded PRPF8 protein is the largest and most highly conserved spliceosome component that plays critical roles in pre-mRNA splicing as core component of precatalytic, catalytic and post-catalytic spliceosomal complexes during the splicing cycle (Achsel et al., 1998; Grainger and Beggs, 2005). PRPF8 protein serves as a scaffold to mediate the assembly of U4/U6.U5 tri-snRNP and positions U2, U5, and U6 snRNAs at splice sites on pre-mRNA substrates. PRPF8 binds to 5′ splice site, branch point and 3′ splice site of pre-mRNA as well as U5 and U6 snRNAs. Immunoprecipitation experiments have revealed the association of PRPF8 with pre-mRNA, lariat intermediate, lariat product, and ligated exons (Luo et al., 1999). A Jab1/MPN domain resides in the C-terminal region of PRPF8, which interacts with the SNRNP200 helicase to regulate U4/U6 snRNA duplex unwinding during B complex activation (Maeder et al., 2009; Mozaffari-Jovin et al., 2012, 2013; Nguyen et al., 2013). In adRP cells, mutant PRPF8 protein displays less effective interaction with SNRNP200, defective splicing site recognition, and U4/U6 unwinding, affecting spliceosome assembly, which contributes to suppression of splicing efficiency (Tanackovic et al., 2011b; Wickramasinghe et al., 2015; Mayerle and Guthrie, 2016). Mutations in *PRPF8* have been implicated in the development of adRP and primary open angle glaucoma (POAG) (Tarttelin et al., 1996; Gamundi et al., 2008; Walia et al., 2008; Towns et al., 2010; Micheal et al., 2018). The co-segregation of *PRPF8* mutations with adRP was first revealed in 2001 (McKie et al., 2001). To date, 64 *PRPF8* mutations have been reported (see footnote 2), including missense/nonsense, small deletion, small insertion, small indel, gross deletion. Notably, most of the mutations cluster in PRPF8 C-terminal region, which contains functional domains that are related to interaction with pre-mRNA 5′ splice site, interaction with the RNA helicase SNRNP200 and GTPase EFTUD2 (HSNU114). Thus, *PRPF8* mutations can lead to multiple defects in spliceosome assembly and activation during pre-mRNA splicing. In contrast to *PRPF31*, both the wild type and mutant alleles in RP13 cell lines with a nonsense mutation displayed comparable expression of *PRPF8*, suggesting that haploinsufficiency is not the cause of *PRPF8*-RP (Gamundi et al., 2008).

## Disease Models for *PRPF*-RPs

Since the first observation of pre-mRNA splicing reaction in Berget et al. (1977), a variety of cellular and animal models have been adopted for the investigation of spliceosome machinery, disease-related splicing defects and therapeutic studies. From single-celled yeast to metazoan *Caenorhabditis elegans* and mammalian systems such as mice and non-human primates, exquisitely designed laboratory models have substantially expanded our knowledge on the mechanisms unpinning RNA splicing. These are discussed as follows:

### Yeast Models

RNA splicing remains highly conserved during evolution. In yeast and metazoans, spliceosome assembly and RNA splicing reaction steps are similar, so the investigations of RNA splicing factors in yeast can be generalized to multicellular eukaryotic organisms including humans (Anderson et al., 1989; Hossain and Johnson, 2014). *Saccharomyces cerevisiae* exists stably as both haploid and diploid cells and two haploid cells mate to form a single diploid cell. Based on this important feature, the yeast two-hybrid system provides a powerful tool for the study of spliceosome conformation and the interaction among components (Liu et al., 2006). In addition to the yeast two-hybrid system, co-immunoprecipitation, isothermal titration calorimetry, biochemical complementation assay, synthetic lethality assay etc. provide additional assays for validation of potential interactions between components of the spliceosome.

Screening of temperature-sensitive yeast mutants using the intron-containing *CRY1* and *ACT1* genes as hybridization probes is widely used for identification of pre-mRNA splicing defects and related splicing factors (Lustig et al., 1986; Warner, 1987; Maddock et al., 1996; Noble and Guthrie, 1996). In *S. cerevisiae* more than 30 gene products have been identified as essential for RNA splicing which are referred to as precursor RNA processing proteins (PRPs) including orthologs of all human pre-mRNA processing factors responsible for RP (Weidenhammer et al., 1996; Faustino and Cooper, 2003).

*PRP3* is the yeast homolog of *PRPF3*. *PRP3* mutant was identified in a screen of temperature-sensitive yeast strains with accumulation of unspliced pre-mRNAs, indicating the blockage of spliceosome assembly due to dysfunction of Prp3 protein (Anthony et al., 1997). The temperature sensitive mutation of the *PRP3* gene was rescued by extra copies of the wild type *PRP4* gene (Hu et al., 1994); however, a double mutant of *PRP4* and *PRP3* exhibited synthetic lethality (Wang et al., 1997). The biochemical complementation and synthetic lethality by *PRP3* and *PRP4* mutations corroborate their interaction in U4/U6 snRNP. The deletion of 5′ stem-loop in U4 snRNA inhibits the Prp4-snRNA interaction and suppresses the formation of U4/U6.U5 tri-snRNP, indicating the important roles of Prp4 protein and this region of the U4 snRNA in spliceosome assembly (Bordonne et al., 1990). Human PRPF6 homolog Prp6 in yeast functions in the same way as its counterpart in mammalian cells to bridge the association between U4/U6 snRNP and U5 snRNP. A temperature-sensitive strain carrying mutations at *PRP6* locus displayed inactivated RNA splicing at 37°C (Abovich et al., 1990). By temperature-sensitive screening of splicing defective yeast strains, *PRPF31* ortholog *PRP31* was identified and shown to be important for cell viability (Maddock et al., 1996). *PRP31* mutant yeast strain is defective in splicing *in vitro* as well as *in vivo*, indicating the direct contribution of Prp31 to splicing pathway (Weidenhammer et al., 1997).

Prp8/PRPF8 is a highly conserved pre-mRNA splicing factor from yeast to man. Yeast two-hybrid system assays have confirmed the interaction between Prp8 and Brr2 (the yeast homolog of the human RNA helicase SNRNP200), which is interrupted by RP mutations in the C-terminus of Prp8 protein (van Nues and Beggs, 2001; Pena et al., 2007). Yeast strains with *PRP8*-depletion, inactivation or introduction of RP-causing mutations display temperature sensitivity and reduced growth rate. The impaired Prp8 function inhibits spliceosome assembly, causing severe splicing defect and abnormal alternative splicing profile (Jia and Sun, 2018). Boon et al. (2007) revealed a U5 snRNP precursor in the cytoplasmic containing Prp8-Aar2 interaction that is converted to Prp8-Brr2 when imported into the nucleus to become mature snRNP complex (Weber et al., 2013). The *PRP8*-RP mutation disrupts the Prp8–Brr2 interaction, thereby reducing the functional U5 snRNP and U4/U6.U5 tri-snRNP formation in the nucleus (Mozaffari-Jovin et al., 2013). Furthermore, high-throughput screening with temperature-sensitive yeast mutant strains in search of therapeutic compounds or target genes/proteins identified Srn1 as a suppressor of the defects caused by several of the *PRP* mutants (Pearson et al., 1982). While Spp81 was identified as suppressor of *PRP8* mutation (Jamieson et al., 1991). And the pharmacological chaperone, 9-*cis* retinal, could partially rescue light-dependent activation of a disease-associated rhodopsin mutation (Pro23His) expressed in yeast (Scott et al., 2019).

Taken together, the yeast system is ideal for the screening of splicing factors, the study of interactions between spliceosome components and the effects of splicing factor mutations on spliceosome assembly and RNA splicing reactions. However, the characteristics of the yeast cells differ significantly from the human retinal cells and are thus unable to fully mimic the PRPF-RP disease phenotypes.

### Zebrafish Models

Being a vertebrate metazoan, zebrafish has major tissues and organs including eyes with similar characteristics to humans, and as such proven to be an excellent model for developmental and disease studies. Adult zebrafish breed approximately every 10 days and can produce 50–300 eggs at a time, providing many experimental specimens in a short period of time. Zebrafish embryos develop externally and are nearly transparent, making microscopic examination easy. With fertilized eggs at the one-cell-stage, genetic modification can be easily achieved through microinjection of DNA or RNA (Zheng et al., 2018). The zebrafish genome has been fully sequenced and shares 70% of genes with human, and more than 80% of the disease-related human genes have their zebrafish counterparts (Howe et al., 2013). RP-associated *prpf* genes have been modulated in zebrafish as animal models for the study of RP pathogenesis and drug screening.

Homozygous *prpf3* knockout is lethal to zebrafish, resulting in death at 4 days post fertilization. Within the knockout embryos, elevated cell death level occurs in the developing eyes. However, no developmental abnormality or retinal degeneration is observed in the heterozygous *prpf3* knockout zebrafish, suggesting that RP18 is caused by toxic gain of function by missense mutations in *prpf3* rather than haploinsufficiency (Amsterdam et al., 2004; Graziotto et al., 2008).

*Prpf4* knockdown in zebrafish results in photoreceptor morphology alteration and degeneration (Linder et al., 2014). The mutation of c.575 G > A (p.R192H) in the zebrafish homolog of *prpf4* gene disrupts the interaction between prpf4 and prpf3 and interferes with the integration of prpf4 into the tri-snRNP and spliceosome (Linder et al., 2014). The introduction of *prpf4* c.944C > T (Pro315Leu) missense mutation in zebrafish leads to systemic deformation of larvae including malformed brains, short trunks, cardiac edema, and curved body axis as well as disrupted photoreceptor inner and outer segment structure and compromised rhodopsin reactivity (Chen et al., 2014). Co-injection of *prpf4* c.944C > T and morpholino oligos (MOs) against *prpf4* in zebrafish significantly increases embryo death rate, indicating that c.944C > T acts as a dominant-negative mutation in the morphant that competes the residual function of wild type zebrafish Prpf4 protein. Notably, several retinal transcripts are downregulated by *prpf4* c.944C > T mutation, implicating the molecular mechanism of RP70 associated with *prpf4* mutation.

Prpf31 protein is essential for zebrafish embryonic survival and eye development. *Prpf31* silencing by morpholino oligonucleotides (MOs) results in severe embryonic malformations and lethality. Knockdown of *prpf31* primarily affects retinal photoreceptor outer segments and impairs visual function showing substantially decreased optokinetic nystagmus (OKN), and in severe cases there are no saccadic movements (Linder et al., 2011). Gene expression analysis reveals that a set of retinal genes, such as *rho*, *gnat2*, and *pde6c*, are selectively downregulated in *prpf31* morphants and this may cause the retina-specific phenotype. Two RP-related mutations in *prpf31*, SP117 (c.769insA) and AD5 (c.1115_1125 del) were introduced into zebrafish (Yin et al., 2011). Both mutations led to instability of the mutant proteins. SP117 (c.769insA) displayed aberrant cytoplasm localization in rod photoreceptors, however rod morphology or numbers were not affected. Injection of SP117 (c.769insA) mutant mRNA into zebrafish embryos neither rescued the *prpf31* morphant phenotype nor increased the degeneration of photoreceptors, suggesting haploinsufficiency could be the underlying mechanism in human RP patients carrying SP117 (c.769insA) mutations. AD5 (c.1115_1125 del) mRNA injection led to embryonic malformations and dose-dependent lethality. Retinas expressing AD5 (c.1115_1125 del) displayed loss of the outer segments, disintegrated nuclei, and increased apoptosis in rod photoreceptors. The wild type *prpf31* mRNA rescued the embryonic lethality induced by AD5 (c.1115_1125 del), however, AD5 (c.1115_1125 del) mutant RNA was not able to rescue the *prpf31* morphant phenotype, but instead aggravated the MO phenotypes, indicating that AD5 acts in a dominant-negative fashion in RP pathogenesis. Expression of phototransduction-related genes were down-regulated in AD5 expressing retinas, including *rho*, *gnat1*, *guk1*, *rcv1*, *calb2*, which display significantly stabilized introns, suggesting the disrupted mRNA splicing by AD5 mutation as a cause of photoreceptor dysfunction.

### Mouse Models

Mice are evolutionarily more like humans, because they are mammals, and therefore normally used as models of human diseases. Homozygous/heterozygous knock-in/knockout mouse models provide platforms for comparative studies to elucidate the pathogenic mechanisms of *Prpf* mutations associated RPs.

Homozygous *Prpf3* and *Prpf31* knockout are lethal to the mouse embryo, while no photoreceptor degeneration or retinal dysfunction is observed with heterozygous knockout mice (Graziotto et al., 2008; Bujakowska et al., 2009). Transgenic mice with *Prpf3^*T*494*M/*^*^+^ and *Prpf8^*H*2309*P/+*^* mutations display late-onset RPE degenerative morphologies, including loss of basal infoldings, accumulation of amorphous deposits beneath the RPE, and vacuolization in the cytoplasm. These RPE abnormalities are more severe in the homozygous *Prpf3*-T494M and *Prpf8*-H2309P mice, suggesting that RP18 and RP13 are caused by the gain-of-function or dominant-negative effect of *Prpf3* and *Prpf8* mutations (Graziotto et al., 2011; Farkas et al., 2014). Such phenotypes are also observed in *Prpf31*^±^ knockout mice. Moreover, transfection of truncated *Prpf31* mutants N371 or N256 into primary mouse retinal culture cells leads to significant apoptosis (Yuan et al., 2005). Thereby, both haploinsufficiency and dominant-negative effects are likely to underly the pathogenic mechanism of *Prpf31* mutations. Daily phagocytosis of shed photoreceptor outer segments (POS) is one of the key functions of RPE (Kevany and Palczewski, 2010). The *Prpf31*^±^ mutant RPE cells show remarkably reduced phagocytosis, demonstrated by declined phagocytic peak intensity and altered diurnal rhythmicity, attenuated phagocytic burst after light onset, and spreading of the phagocytic peak time. The adhesion between the RPE apical microvilli and the POS is significantly reduced, suggesting disturbance of the integral function of the neural retina and RPE. Additionally, *Prpf* mutant mouse models display mis-localization of the major phagocytic receptors avβ5 integrin, MerTK and signaling protein FAK as well as MerTK ligand GAS6, both located on the apical surface of wild type RPE (Finnemann et al., 1997; Finnemann, 2003; Mao and Finnemann, 2012). Despite the morphological changes and functional deficiencies in mutant RPE, the photoreceptor cells in the mutant mice appear to be normal in histological and ultrastructural analyses and only at 24 months, the *Prpf3*^*T*494*M*^ mice show decreased maximal rod a- and b-waves, but with no significant changes in cone b-wave. These findings suggest that RPE is the primary retinal cell type affected by *Prpf* mutations and the photoreceptor dysfunction is secondary to RPE impairment. Co-transfection of *Prpf31* mutant expressing plasmids with *Rho* intron 3 minigene led to inhibition of *Rho* gene splicing, decreased expression of rhodopsin and apoptosis of rhodopsin positive retinal cells, corroborating the disturbed pre-mRNA splicing of retinal functional genes by *Prpf31* mutation. Application of caspase inhibitor Baf substantially improved the viability of *Prpf31* mutant-expressing retinal cells, implicating its potential therapeutic effect for RP treatment (Yuan et al., 2005).

In a heterozygous knock-in mouse model carrying *Prpf31*^*p.A*216*P*^ mutation, no photoreceptor cell death or increased oxidative stress was observed up to 18 months (Bujakowska et al., 2009; Valdes-Sanchez et al., 2019). In the wild type RPE cells, most of PRPF31 protein is localized inside the nucleus, while the mutant PRPF31 displayed cytoplasmic localization in the form of insoluble aggregates. The wild type PRPF31 protein was also recruited to the insoluble cytoplasmic aggregates by the overexpressed PRPF31 mutant protein, thus depleting it from the nucleus (Valdes-Sanchez et al., 2019). In this way, the *Prpf31*^*p.A*216*P*^ mutation exerts both dominant-negative and haploinsufficiency effects, leading to RPE defects. *Prpf31*^*p.A*216*P/+*^ knock-in mice display RPE degeneration with drusen-like deposits, lipofuscin accumulation, vacuolization of RPE, atrophy of basal infoldings and thickening of Bruch’s membrane, which resembles the features of age-related macular degeneration (AMD). A- and b-waves were not affected by *Prpf31* mutation, but c-wave defects were observed, which is consistent with RPE degeneration. Thousand and thirty-three differential genes were identified in *Prpf31*^*A*216*P/+*^ mice, including the chaperone binding proteins. The upregulation of heat shock protein family A (*Hsp70*) member 4 like gene (*Hspa4l*) in the mutant mice was observed. Moreover, Hspa4l colocalized with PRPF31 mutant aggregates in the RPE cytoplasm, indicating activation of the unfolded protein response (UPR) to facilitate folding of the mutant protein and/or other mis-spliced isoforms. By alternative splicing analysis, more than 10% of all the genes were found differentially spliced including *Prpf31* itself and genes involved in retinal degeneration, such as inflammation, oxidative stress, retinol metabolism (*Abca4*), ciliogenesis (*Bbs1*, *Bbs4*, *Bbs5*, *Bbs7*, *Bbs9*) and apoptosis.

It is not known why *Prpf* mutant mice show only RPE degeneration rather than photoreceptor cell death, which is the key phenotype of human PRPF-RPs. The higher level of *PRPF31* expression in RPE cells than in the neuroretina may partly explain the phenomenon (Valdes-Sanchez et al., 2019). Alternatively, the presence of mutant *Prpf31* selectively in the RPE cells but not in photoreceptors, may confer a dominant negative response in RPE cells, which is probably more severe than haplo-insufficiency observed in photoreceptors (Buskin et al., 2018). More importantly, the phenotypic variation can be caused by the species difference between mouse and human. There are fundamental structural and functional differences between rodent and human retina. The lifespan of mice may be too short to recapitulate the RP manifestation of photoreceptor degeneration.

### Human iPSC Models

A variety of cell lines have been applied to study the pathological effects of *PRPF* mutations, including the widely used ARPE-19 as a model of native human RPE cell (Dunn et al., 1996). However, immortalized cell lines do not recapitulate certain *in vivo* features, making it difficult to represent the complex *in vivo* scenario of RP pathogenesis.

The advent of human induced pluripotent stem cells (hiPSCs) technology offers a novel strategy for *in vitro* modeling of human diseases (Takahashi and Yamanaka, 2006; Takahashi et al., 2007; Yu et al., 2007). By transduction of Yamanaka factors Oct3/4, Sox2, Klf4, c-Myc, patient-derived somatic cells are converted into pluripotent, embryonic-like state with the capacity of unlimited self-renewal and multilineage differentiation, enabling the rapid establishment of disease-specific cell models. These models are widely used for the studies of disease mechanisms, toxicological assay, drug screening, and cell replacement therapies (Artero Castro et al., 2018).

For *in vitro* modeling of human retinal diseases, it is important to incorporate the diversity of all retinal cell types that can be maintained stably in culture for prolonged periods of time. Several differentiation protocols have been established for generation of RPE, photoreceptor-like cells as well as retinal organoids containing all retinal cell types (Lamba et al., 2010; Kokkinaki et al., 2011; Nakano et al., 2012; Zhong et al., 2014; Mellough et al., 2015). In our group, an optimized protocol for retinal organoid differentiation has been created through statistical design of experiment (DoE) methodology, to efficiently induce laminated, physiologically functional, and light responsive retinal organoids on a large scale (Hallam et al., 2018).

For modeling of *PRPF*-RP, hiPSCs reprogramming from RP patient fibroblasts carrying *PRPF8* missense mutations has been reported (Lukovic et al., 2017; Foltz et al., 2018). The established hiPSCs differentiated into RPE cells, showed apical basal polarity and phagocytosis of POS in the same manner as wild type cells. Terray et al. (2017) established an induced pluripotent stem (hiPSC) cell line from dermal fibroblasts of an asymptomatic patient with dominant *PRPF31* mutation. The reprogrammed hiPSC contained the expected c.709-734dup substitution in exon 8 of *PRPF31*, expressed the expected pluripotency markers, displayed *in vivo* differentiation potential to the three germ layers and had normal karyotype, but deeper studies into RP pathogenesis were not carried out (Terray et al., 2017). In our previous work, dermal fibroblasts from RP11 patients with *PRPF31* c.1115_1125del11 and c.522_527 + 10del heterozygous mutations were reprogrammed to hiPSCs and differentiated into RPE and retinal organoids (Buskin et al., 2018). A combination of molecular, phenotypic, and biochemical assays showed the RP11-RPE to be the most affected retinal cell type. Interestingly, the RP11-RPE and photoreceptors, but not the hiPSCs or fibroblasts, were characterized by a global spliceosome dysregulation, matching with the retinal specific phenotype of RP11. The RP-11 RPE cells displayed a variety of cellular phenotypes ranging from loss of apical – basal polarity, impaired tight epithelial barrier, shorter and fewer microvilli, defective phagocytotic function and the presence of large basal deposits. Ultrastructural analysis revealed that RP11-photoreceptors had more apoptotic nuclei and stress vacuoles compared to the controls. RP11 organoids also showed reduced responses to neurotransmitter GABA suggesting the impaired neural networks in RP11 patients. Compared to controls, RP11-RPE and retinal organoids displayed decreased *PRPF31* expression and compromised splicing ability. RP11-RPE cells but not photoreceptors, were characterized by the presence of mutant *PRPF31* transcript, which may indicate a dominant negative function in these cells and may explain the more severe phenotype. Both cilia incidence and cilia length were significantly reduced in RP11 – RPE and photoreceptors. Our work showed that the splicing of multiple ciliary genes was affected, thus it is likely that ciliary phenotype is a direct consequence of retinal specific spliceosome dysregulation. While the role of cilia in photoreceptors is well-established, recent studies suggest that cilia function is also important for RPE maturation and function (May-Simera et al., 2018), hence, cilia abnormalities in both RP11-RPE and photoreceptors are likely to worsen the primary cellular phenotypes due to splicing mis regulation.

Using CRISPR/Cas9-mediated gene-editing, the mutation can be corrected to produce isogenic-matched control hiPSC lines. Correction via CRISPR/Cas9 genome editing of patient hiPSCs was carried out and hiPSC-RPE and retinal organoids were used to investigate if *in situ* gene editing could ameliorate/reverse the cellular phenotypes. Indeed, the gene edited hiPSC derived RPE and photoreceptors were characterized by longer and more frequent cilia with well-aligned axonemal microtubules and restored splicing ability. Moreover, the impaired RPE apical-basal polarity and phagocytic capacity were restored after CRISPR/Cas9 correction, suggesting that gene editing provides a promising approach for RP11 treatment.

In summary, all the RP-*PRPFs* have been investigated utilizing a variety of modeling systems. Heterozygous *Prpf3* knockout mice show no photoreceptor degeneration, while RPE degenerative abnormalities are induced in *Prpf3* mutation transgenic animals, indicating that RP18 is caused by the dominant-negative or gain-of-function effect of *Prpf3* mutations. Similar phenotype is observed in *Prpf8* mutation transgenic mice. *prpf4* knockdown in zebrafish results in retinal degeneration. Injection of *prpf4*^*p. Pro*315*Leu*^ and morpholino oligos (MOs) against *prpf4* in zebrafish triggers retinal deformities and increase of embryo death rate, indicating that mutations of *prpf4* cause RP via haploinsufficiency and dominant-negative effects. Disruption of *PRP6* genes results in yeast lethality. *PRPF6* knockdown in HeLa SS6 cells significantly suppresses cell growth. However, it is unclear whether *PRPF6* mutations act as haploinsufficient or dominant-negative elements. In zebrafish, *prpf31* knockdown predominantly damages the retinal photoreceptor outer segments and impairs visual function, which is deteriorated by AD5 (c.1115_1125 del) mutant RNA injection. Therefore, both loss-of-function and dominant-negative effects are involved in the pathogenic process of *prpf31* mutations. Of note, introduction of *Prpf31*^*p.Ala*216*Pro*^ mutation into mice model has no detrimental effect on the retina, suggesting that various mutations may have distinct mechanisms. In addition, all modeling systems have advantages and disadvantages (Supplementary Table 3). A full investigation and analysis using multiple models would be a great strategy to gain a thorough understanding of the mechanisms underlying a certain disease.

## Impacts of *PRPF* Mutations on RNA Splicing and Gene Expression Profile

RNA splicing is at the intersection of transcription and translation, which explicitly controls both transcript abundance and diversity. Mutations of spliceosome components can have trans-acting effects on RNA splicing, leading to aberrant transcript products and resultant genetic diseases. By microarray profiling of peripheral blood samples from RP13 patient bearing the p.H2309R mutation in *PRPF8*, transcriptome-wide splicing defects were identified, affecting approximately 20% of exons. Greater intron inclusion in mature transcripts was observed in mutant compared to controls. Flanked by long introns, the differentially spliced exons were shorter than average, AT rich and located toward the 5′ end of the gene (Korir et al., 2014). PRPF8 protein is involved in both the major U2-dependent splicing (snRNPs: U1, U2, U4/U6, and U5) and the minor U12-dependent splicing (snRNPs: U11, U12, U4atac, U5, and U6atac), as opposed to other pre-mRNA splicing factors that participate in U2-dependent spliceosome only (Luo et al., 1999), so *PRPF8* mutant may have distinct effects on splicing that are different from other *PRPFs*. Consistently, the accumulation of aberrantly spliced transcripts retaining both U2-and U12-introns was observed in the zebrafish mutant Cephalophonus (cph) bearing an early premature STOP codon in *prpf8* (Keightley et al., 2013).

In retinal cells generated via the hiPSC differentiation, high level of transcripts with retained introns and alternative 3′ splice sites were identified in *PRPF31* mutant organoids and RPE cells (Buskin et al., 2018). The top category of mis-spliced genes affected by *PRPF* mutations was pre-mRNA and alternative mRNA splicing via the spliceosome, including spliceosome assembly, formation of the U4/U6 snRNP, 3′-end processing of pre-mRNAs, association of U2 snRNP with pre-mRNA and 5′-splice site selection (Buskin et al., 2018). These results were corroborated by molecular analysis of retinas of *Prpf31*^±^ mouse model (Buskin et al., 2018) and the human organotypic retinal cultures with RNAi mediated knockdown of *PRPF31*, which also displayed mis-splicing of genes involved in RNA processing (*PRPF3, PRPF8, PRPF4*, and *PRPF19*) (Azizzadeh Pormehr et al., 2020). In addition to genes encoding spliceosome component proteins, a substantial decrease in snRNAs including U2, U4, U5, U6 was also observed in lymphoblast cell lines from RP patients with mutations in *PRPF3*, *PRPF8*, and *PRPF31* (Tanackovic et al., 2011b). These data indicate that disrupted alternative splicing programs in RP worsen defects in splicing, and in turn disrupt biological processes outlined below that trigger the unusual cellular phenotypes observed in RP.

Phototransduction is one of the most predominant groups of differentially spliced genes associated with retinal function that are affected by PRPF mutations/depletion. *PRPF31* knockdown in human organotypic retinal cultures caused mis-splicing of genes involved in phototransduction, including *RHO*, *GNAT1*, *GNAT2*, *ROM1*, *PDE6A*, and *PDE6B*, all of which have been linked to inherited retinal diseases (IRDs) (Sakuma et al., 1995; Kohl et al., 2002; Naeem et al., 2012; Cheng et al., 2016; Wang et al., 2018; Azizzadeh Pormehr et al., 2020). *RHO* has been verified to be the splicing target for PRPF31 as a major RP-associated gene. The expression of mutant *PRPF31* in primary mouse retinal cells inhibited *RHO* splicing, leading to a significant reduction in *RHO* expression and cell death (Yuan et al., 2005). Rhodopsin is a prototypic member of the G-protein-coupled receptor family mediating phototransduction cascade initiated by the isomerization of 11-*cis*-retinal. Loss-of-function *RHO* mutations alone are unlikely to cause adRP, instead, gain-of-function and dominant-negative mutations appear to be the main pathogenic reasons due to unfolded-protein response, aggregation and inclusion formation, structural instability etc. (Mendes et al., 2005). Our previous work revealed exon 21 skipping in *PDE6B* in retinal organoids carrying *PRPF31* mutations (Buskin et al., 2018). PDE6 complex controls intracellular cGMP levels by the hydrolyzation of cGMP in response to light activation and has a major role in the phototransduction (Qureshi et al., 2018). The impaired phototransduction due to the mis-splicing of key functional proteins may represent a critical mechanism underlying *PRPF*-RPs.

In line with the decreased cilia length, cilia incidence and cilia malformation in *PRPF31* mutant RPE cells and retinal organoids, the splicing and expression of important genes related to cilium assembly, cilium organization, microtubule organizing center and centrosome were affected by *PRPF* mutations (Buskin et al., 2018). Most of the affected genes such as *IFT27*, *BBS4* and *ARL6* are critical for cargo recruitment and transportation to support ciliogenesis and maintenance. The photoreceptor outer segment is a special subcellular organelle derived from primary cilium that plays important roles in phototransduction, visual cycle and RPE phagocytosis (Molday and Moritz, 2015). Mutations in these primary cilium component genes are associated with retinal degenerative diseases including RP (Wheway et al., 2014; Schaefer et al., 2019). Two photoreceptor disk morphogenesis related genes peripherin (*PRPH2*) and retinal fascin (*FSCN2*) are significantly suppressed by *PRPF* mutations as well (Mordes et al., 2007). Both *PRPH2* and *FSCN2* are also photoreceptor-specific genes. *PRPH2* encodes a cell surface glycoprotein localized along the rims of rod and cone photoreceptors. As an adhesion molecule, PRPH2 protein is involved in stabilizing and compacting the outer segment disks or in preserving the rim curvature (Travis et al., 1991; Arikawa et al., 1992). *Prph2*^–/–^ knockout mice showed complete absence of outer segment (Chakraborty et al., 2014) and heterozygous mutations in the *PRPH2* gene are a common cause of adRP (Keen and Inglehearn, 1996). FSCN2 protein is a fascin family member that crosslinks F-actin into filamentous bundles within dynamic cell extensions and plays important roles in photoreceptor disk morphogenesis (Saishin et al., 1997; Tubb et al., 2000). In mice models carrying *Fscn2* mutations of c.208delG or replacement of exon 1, *Fscn2* expression is substantially suppressed, along with aberrant outer segment disks, progressive photoreceptor degeneration and depressed rod and cone electroretinograms (ERGs), indicating that haploinsufficiency of the *Fscn2* gene may impede maintenance and/or elongation of the OS disks, resulting in photoreceptor degeneration (Yokokura et al., 2005). The c.208delG mutation in the *FSCN2* gene was identified in 14 individuals with adRP from four unrelated Japanese families, and it cosegregated with the phenotype in all four pedigrees, implying an association to RP (Wada et al., 2001). However, another study on Chinese families came to the opposite conclusion, revealing that the c.208delG mutation has a negative cosegregation with retinal degeneration and that this mutation was identified in normal family members and control subjects (Zhang et al., 2007). It is unclear whether ethnicity or other factors influence the sensitivity to this mutation. Further research with a bigger sample size representing individuals from multiple ethnic backgrounds may help to address the question. Taken together, mis-splicing of important genes involved in photoreceptor functions are most likely to contribute to the multi-faceted *PRPF*-driven RP pathogenesis.

Other significantly affected genes in *PRPF* mutated human cells include those involved in actin cytoskeleton, axon terminals, lysosome and endosomal processes, focal adhesion, cell-substrate junctions, and extracellular matrix organization, signaling pathways, apoptosis, inflammation, UPR etc. (Supplementary Table 2). Although these general functions are not directly related to RP pathogenesis, they may be affected to a greater extent in the retina due to the more severe dysregulation of RNA splicing machinery, thereby facilitating the deterioration of retinal abnormalities and photoreceptor degeneration.

## Beyond RNA Splicing

**FIGURE 3:**
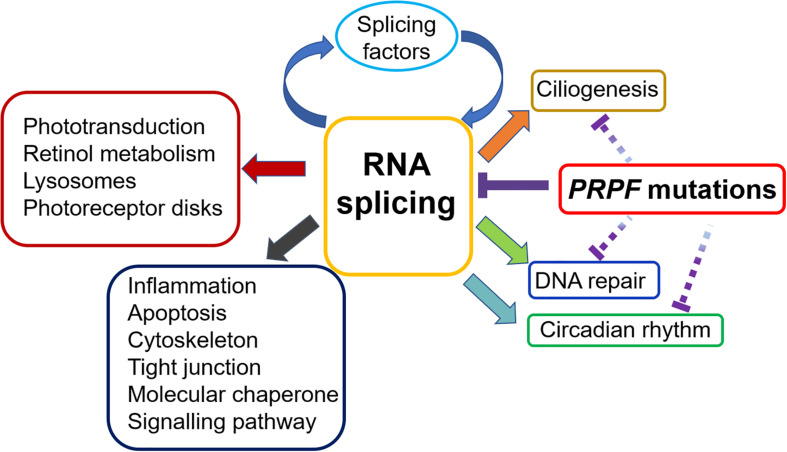
Impacts of *PRPF* mutations on various biological processes in the retina. RP-related *PRPF* mutations primarily result in disrupted spliceosome assembly and defective RNA splicing, leading to subsequent alteration of splicing profile. Many of the affected genes are involved in important retinal functions, including phototransduction, retinol metabolism and photoreceptor disk, etc. More mis-spliced genes cluster in general biological processes, such as inflammation, apoptosis cytoskeleton, signalling pathway, etc. Noteworthy, splicing factors are enriched in the top category of mis-spliced genes affected by *PRPF* mutations, indicating a feedback loop that deteriorates the RNA splicing defect in retina. Furthermore, emerging evidence suggests that *PRPF* mutations may contribute to RP development via mechanisms beyond RNA splicing. The interrupted lines indicate likely direct effects of *PRPF* mutations on ciliogenesis, circadian rhythm and DNA repair.

In recent years, PRPF function implications beyond RNA splicing have attracted increased attentions. In the section below, we will review the role of PRPFs in ciliogenesis, circadian rhythm and DNA damage and repair (Figure 3).

### PRPFs and Ciliogenesis

Photoreceptors contain a primary cilium (known as the connecting cilium) that plays a critical role in transporting cargo from inner segment to the outer segments (Adams et al., 2007). By whole genome siRNA-based reverse genetics screen, *PRPF6*, *PRPF8*, and *PRPF31* were identified as candidate genes involved in biogenesis and/or maintenance of the primary cilium. Moreover, these PRPF proteins were localized to apical inner segment, basal body complex and apical connecting cilium of photoreceptor cells (Johnson and Malicki, 2019). In accordance, fibroblasts from RP11 families carrying the heterozygous *PRPF31* frame-shift mutation c.1115_1125del showed significantly shorter and fewer cilia (Wheway et al., 2015). In RP11-iPSC-derived retinal organoids and RPE cells harboring two different *PRPF31* mutations, both cilia incidence and cilia length were significantly reduced compared to wild type controls. Serial block face scanning electron microscopy (SBFSEM) analysis revealed shorter, abnormal, bulbous cilia with misaligned microtubules in RP11-RPE cells. By structured illumination microscopy (SIM) analysis, RP11-RPE were characterized by mislocalization of IFT88 protein to the ciliary tip and the transition zone (TZ) proteins CC2D2A and RPGRIP1L were either entirely excluded from the TZ or mis localized from the TZ into the ciliary axoneme (Buskin et al., 2018).

Centrosomes are dynamic organelles composed of bound pair of microtubule-based centrioles, which serve as basal bodies required for the formation of primary cilia at cell surface. Two studies of the centrosomal proteome, identified PRPF6 and PRPF8 as centrosomal proteins (Andersen et al., 2003; Jakobsen et al., 2011). Mutations in the cilia-centrosomal protein gens Retinitis Pigmentosa GTPase Regulator (*RPGR*) and *FAM161A* are the major causes of X-linked and arRP respectively (He et al., 2008; Bandah-Rozenfeld et al., 2010; Langmann et al., 2010). These findings suggest that, independent of their nuclear function in splicing, PRPFs could serve as important cilia component proteins participating in ciliogenesis and maintenance of photoreceptor outer segment. It is currently unknown whether their cilia-specific localization relates to their splicing function, or whether PRPFs have novel functions that affect cilia formation during retinal ontogenesis.

### PRPF and Circadian Rhythm

Circadian rhythm is an endogenous body clock that drives the oscillation of various physiological functions such as hormone production, digestion, blood pressure, body temperature etc. (Patke et al., 2020). The retinal ganglion cells (RGCs) receive and relay light signal to the circadian pacemaker in the suprachiasmatic nuclei of the hypothalamus (SCN), which subsequently transfer phase information to peripheral oscillators. Circadian rhythms are controlled by autoregulatory transcription factors including *Clock, Bmal1, Per, Cry, Rev-Erb*, and *Ror* that form positive and negative feedback-loops for the regulating system (Ko and Takahashi, 2006).

An intrinsic extra-SCN circadian clock system is present in the retina, which controls many aspects of retinal biological processes including gene expression, signaling, metabolism, retinal electrical responses to light, synthesis and secretion of neurotransmitters, photoreceptor outer segment shedding etc. (McMahon et al., 2014). Periodic phagocytosis by RPE of spent POS is one of the most remarkable retinal circadian processes regulated by circadian oscillator and the daily light-dark cycle (Grace et al., 1999). Both photoreceptors and RPE cells have independent circadian oscillators that regulate the rhythm of renewal, shedding and phagocytosis of POS (Baba et al., 2010; Ko, 2020). In this regard, the circadian clocks of photoreceptors and RPE must closely synchronize to ensure the efficient clearance of the spent POS, which is critical for maintaining retinal health. Retinal cells show extensive expression of core clock genes such as *PER1*, *BMAL1*, *CLOCK*, *CRY1*, and *CRY2* that are under circadian control themselves (Tosini and Fukuhara, 2002; Liu et al., 2012; Hiragaki et al., 2014) and are responsible for the regulation of circadian gene expressions in the retina. Some important photoreceptor-specific genes are also under circadian control, such as light-sensitive receptor proteins opsin and rhodopsin, cyclic nucleotide–gated channels (CNGC), L-type calcium channels (LTCCs) and retinal Crystallins that act as chaperone proteins and play critical roles in photoreceptor protection (Ko, 2020). In *Bmal1* knockout mice, a large fraction of these rhythmic genes showed non-rhythmic expression or reduced amplitude due to circadian dysregulation, and visual function and photoreceptor viability were impaired during aging (Baba et al., 2018a).

Melatonin and Dopamine are two key circadian elements in the retina that reciprocally antagonize each other (Tosini et al., 2008) and play an important role in photoreceptor survival. During the night, photoreceptors release melatonin that inhibits the release of dopamine by dopaminergic amacrine cells (DA). During the day, DA-released dopamine prevents the melatonin synthesis and release from the photoreceptors (Tosini and Dirden, 2000; Doyle et al., 2002). Melatonin signaling was shown to impact the viability of photoreceptor via the MT1 melatonin receptor. In *Mti*^–/–^ mice, the daily rhythm of ERG a- and b-wave amplitudes was lacking, and significant loss of photoreceptor was detected (Baba et al., 2009). On the other hand, dopamine was reported to play a critical role in photoreceptor degeneration. In *rd* mice, a well-established RP model, antagonists for D1-/D2- dopamine receptors or dopamine depletion improve photoreceptor survival, while addition of a dopamine agonist in dopamine-depleted organ cultures induces photoreceptor degeneration (Ogilvie and Speck, 2002). In line with the rhythmic secretion of dopamine and melatonin, their widespread receptors are also under circadian control in the retina to ensure the integration and coordination of the clock signal system (Wiechmann and Smith, 2001; Nir et al., 2002; Baba et al., 2009; Ikegami et al., 2009).

Recent studies have linked mutations in *PRPFs* to the circadian dysregulation in retina. Mouse model carrying a homozygous H2309P mutation in *PRPF8* showed a lengthened period of the circadian wheel-running activity rhythm as well as dampened daily cycling rhythm of core circadian clock gene transcripts *PER2*, *Rev-Erb*α and *TIM* in retina (Shakhmantsir et al., 2020). Likewise, downregulation of *PRPF31* in a human osteosarcoma cells (U2OS) culture model led to circadian period lengthening, corroborating the regulatory effect of PRPFs on circadian rhythm. In another study, primary RPE cells from *Prpf3* ^*T*494*M/T*494*M*^, *Prpf8* ^*H*2309*P/H*2309*P*^, and *Prpf31*^±^ mice displayed decreased phagocytosis of POS and elongated phagocytic peak time, suggesting the defective diurnal rhythmicity of phagocytosis (Farkas et al., 2014). *Prpf4* knockdown in Drosophila tim^+^ clock neurons led to a significant intron retention in *tim* mRNA, decrease of Tim protein level, delayed nuclear accumulation of Per and Tim and lengthened free-running circadian periods. Strikingly, clock-specific knockdown of *prp3, prp8*, and *prp31* resulted in lengthening of free-running circadian period and complete arrhythmicity (Shakhmantsir et al., 2018). The impacts of *PRPF* mutations/downregulation on circadian period in different species suggest that PRPF factors are broad and evolutionarily conserved circadian regulators.

In our previous work on retinal organoids derived from *PRPF31* mutant iPSCs, intron retention and alternative 5′ splice sites were identified in the transcripts of circadian gene *PER3*, implicating the impacts of RNA mis-splicing on retinal circadian system (Buskin et al., 2018).

Emerging evidence suggests that circadian disruption is associated with the development of retinal disorders. RP patients were found to have increased daytime somnolence, diminished alertness, and poorer quality with more disturbed night-time sleep than their normally sighted counterparts, suggesting an impaired circadian clock (Ionescu et al., 2001). On the flip side of the story, knockout mice of circadian clock genes (*Bmal1^–/–^, Per^1–/–^, and Per^2–/–^*) show reduced photoreceptor viability during aging and scattered retinal deformation by fundus inspection (Bunger et al., 2000; Ait-Hmyed et al., 2013; Baba et al., 2018b). Furthermore, *Rev-Erba*^–/–^ mice showed increased light sensitivity, represented by shortened latencies of both ERG a- and b-wave, modified scotopic and photopic b-wave and scotopic threshold responses, and increased pupillary constriction (Ait-Hmyed Hakkari et al., 2016). Together these reports indicate a close link between photoreceptor viability and regulation of circadian rhythm. PRPFs may be implicated in these synergistic regulations by way of regulating the splicing of circadian clock genes, however, a secondary impact on circadian clock genes as result of overall retinal degeneration caused by *PRPF* mutations cannot be excluded. A deeper exploration of the hypothesized *PRPFs*-circadian system-RP model may provide a novel view to understand the mechanisms underlying *PRPF* mutation-associated RP.

### *PRPFs* and DNA Damage Repair

As a light sensor, the retina is constantly exposed to ultraviolet radiation. During the vertebrate visual cycle, 11-*cis*-retinal bleaching by light leads to the generation of free radical and oxidative stress (Roehlecke et al., 2009, 2011; Organisciak and Vaughan, 2010; Punzo et al., 2012; Kiser et al., 2014; Geiger et al., 2015). Retina is one of the most metabolically active tissues, which implies greater oxidative harm by reactive oxygen species (ROS), a natural by-product of mitochondrial metabolism. Oxidative stress and metabolic dysregulation have been proposed to be associated with retinal degeneration and vision loss (Shen et al., 2005; Punzo et al., 2012) and increased oxidative damage to proteins, lipids and DNA by ROS was found during the course of RP (Domènech and Marfany, 2020).

Maintaining genomic integrity is important for proper cell function and the fidelity of genome transfer to progeny. However, DNA can be damaged by environmental factors such as UV light, ionizing radiation, and endogenous metabolic products such as ROS. DNA lesions caused by detrimental factors comprise single strand break (SSB), double strand break (DSB), intra- and inter-strand cross-links, and DNA adducts etc. Cells have evolved an elaborate DNA damage response (DDR) system to deal with the fundamental problem of genomic erosion, including DNA repair, chromatin remodeling, replication, cell cycle checkpoint, damage tolerance, energy control, and programmed cell death. Three PI3-kinase protein kinases – ATM (Ataxia Telangiectasia mutated), ATR (ATM related), and DNA PK (DNA-dependent protein kinase) are at the mammalian DDR apex (Kolodner et al., 2002; Harper and Elledge, 2007; Menolfi and Zha, 2020). DSBs are primarily sensed by the MRN complex (consisting of Mre11, Rad50, and Nbs1) that recruits ATM to the DNA damage sites. RPA binds to single strand DNA and recruits ATR via its association with ATRIP. The activated ATM/ATR kinases activate other kinases, including CHK1 and CHK2 (Ciccia and Elledge, 2010). Ku70/80 is another DSB sensor that recruits DNA-PK, which plays important roles in non-homologous end joining (NHEJ), telomere maintenance and induction of apoptosis. Three basic DSB repair mechanisms exist: homologous recombination-mediated DNA repair (HR), NHEJ, and single-strand annealing.

Emerging evidence has shown an important role for RNA splicing factors in the cellular response to DNA damage. By genome-wide siRNA screening, hundreds of genes were identified, whose down-regulation led to increased DNA damage, as monitored by the phosphorylation of H2AX (γH2AX), an early mark of DNA damage (Jimeno et al., 2002). Notably, genes involved in mRNA processing represented the most significantly enriched category, including *PRPF3, PRPF4, PRPF8, PRPF31, PRPF19, SART1, SF3A1* etc. Consistently, knockdown of most splicing factors induced a significant increase in cells with multiple 53BP1 foci. Knockdown of splicing factors *CDC40* and *SKIIP* also caused a defective G2/M checkpoint in response to DNA damage (Paulsen et al., 2009).

RNA splicing machinery responds to DNA damage at multiple levels including splicing of DNA repair genes, DNA damage recognition, signaling and repair, RNA-DNA hybrid formation and genome stability etc. (Wickramasinghe and Venkitaraman, 2016). In *prpf31* knockout zebrafish, retinal progenitor cells (RPCs) displayed significant mitotic arrest and DNA damage, which was rescued by the wild type human PRPF31. Bioinformatic analysis revealed that deletion of *prpf31* primarily caused the skipping of exons with a weak 5′ splicing site. Moreover differentially spliced genes were predominantly enriched in GO categories of DNA repair and mitotic progression (Li et al., 2021). This is consistent with our findings in *Prpf31*^±^ mouse retina (Buskin et al., 2018) and suggests that PRPFs may affect DDR via splicing of important genes implicated in DNA repair and cell cycle regulation.

The post-translational modifications of splicing factors have been correlated with DDR, reflecting the initial steps of a concerted process that coordinates the response of RNA splicing to DNA damage. Following HEK 293 cell treatment with ionizing radiation (IR), more than 700 proteins became phosphorylated at ATM/ATR consensus sites. Splicing factors PRPF3, PRPF4, and SART3 were included in the list, suggesting that they were targets for the DDR kinases (Matsuoka et al., 2007). Proteomics analysis of site-specific phosphorylation changes of nuclear proteins after ionizing radiation in B-lymphocyte cells also revealed dephosphorylation of PRPF4 on 18 sites, indicating the importance of controlling RNA processing at the onset of the DDR (Bennetzen et al., 2010). Prp19 complex (Prp19C) or NineTeen Complex (NTC), is a crucial component of spliceosomes that regulates the formation of U4/U6.U5 tri-snRNP complex and is involved in shaping the active site of the spliceosome (Hogg et al., 2010; Song et al., 2010; Fica and Nagai, 2017). Human Prp19C consists of the U-box E3 ligase Prp19 homotetramer complex, CDC5L, SPF27, PRL1, CTNNBL1, AD002, and Hsp73. Upon DNA damage, Prp19C acts as a sensor of DNA damage and directly binds RPA bound to ssDNA, then ubiquitylates RPA to trigger the recruitment of ATRIP and activation of ATR(Marechal et al., 2014; Shkreta and Chabot, 2015; de Moura et al., 2018).

During transcription, newly transcribed RNA anneals to the negatively supercoiled DNA to form a stable RNA–DNA hybrid structure known as R-loop. The exposed non-transcribed single-strand DNA is vulnerable to DNA damage. Deficiency in RNA splicing or defects in mRNA biogenesis can lead to increased R-loop formation and subsequent DNA breaks, transcription and replication stall, and genomic instability (Wongsurawat et al., 2012). In *PRPF31* knockdown RPE-1 cells and primary cells from the stromal vascular fraction (SVF) of *Prpf31*-deficient mouse models (*Prpf31*^±^ and *Prpf31*^+/A216*P*^), significantly increased γH2AX and 53BP1 foci were observed. These were decreased upon overexpression of active RNaseH1, indicating the role of R-loops in genomic instability caused by *Prpf31* depletion (Bhatia et al., 2018). Prp19C is involved in the transcription-coupled repair (TCR) pathway, recruiting the THO/TREX complex to transcribed genes, which is important to prevent the formation of R-loops and genome instability (Chanarat et al., 2011). In accordance with this, *PRP19* mutant *S. cerevisiae* display increased sensitivity to the DNA damaging reagent psoralen as well as other inter-strand cross-link (ICL) inducing reagents (Henriques et al., 1989). In human MRC5 and Hela cells, *PRP19* is significantly up-regulated in response to DNA damage and *PRP19* knockdown results in accumulation of double-strand breaks (DSBs), apoptosis and decreased cell survival after DNA damage induced by γ-radiation (Mahajan and Mitchell, 2003).

RNA splicing is essential for the homologous recombination (HR) repair of chromosomal breaks, which is critical for the stability of the genome. Genome-wide screening studies have revealed the importance of RNA splicing factors (e.g., RBMX and U2 snRNP factor SNRPA1) as positive regulators of HR (Adamson et al., 2012; Tanikawa et al., 2016). To evaluate the influence of the RNA splicing factor PRPF8 on HR, U2OS cells were treated with si*PRPF8* and specific defect in homology-directed repair (HDR), single strand annealing (SSA) and end resection as well as R-loop accumulation were observed. *PRPF8* knockdown caused a significant reduction in foci of DNA repair protein BRCA1, indicating that PRPF8 protein is important for BRCA1 foci formation. Depletion of p53-binding protein 1 (53BP1) suppressed the HR defect induced by both *BRCA1* loss and *PRPF8* knockdown, suggesting that PRPF8 functions during HR at least in part via facilitating BRCA1 function (Onyango et al., 2017).

The retina-specific intensive oxidative stress associated DNA damage and the essential role of RNA splicing factors in DNA repair and maintaining genome stability indicate that the compromised DNA repair machinery due to *PRPF* mutation in RP may not be sufficient to meet the high demand in retina, hence leading to genome instability and retinal degeneration. Together, the roles of PRPFs in ciliogenesis/outer segment shedding, circadian rhythm, and DNA damage repair, indicate their critical functions beyond mRNA processing which may or may not be linked directly to PRPF’s role in splicing, offering new insight into the pathway of PRPF-RP pathogenesis.

## Therapeutic Strategies for *PRPF*-RP

To date there is no cure for RP and therapeutic options are limited. Aside from reduction in light exposure, dietetic interventions have been proposed to benefit RP patients, slowing disease progression (Scholl et al., 2016). For example, RP patients taking high dosage vitamin A showed slowed declines in ERG amplitudes and delayed loss of visual field. Accordingly, daily intake of vitamin A is recommended to adults with RP in the early- or mid-stages (Miggiano and Falsini, 2004). In non-smoking RP patients taking vitamin A, supplementation with lutein 12 mg per day reduced midperipheral visual field deficit on average (Berson et al., 2010). Individuals with the highest DHA (Docosahexaenoic acid) levels in red blood cells displayed the slowest rate of retinal degeneration and people having DHA supplemented diet (1.4 g per week) showed a 40–50% slower visual field loss compared to controls (Berson et al., 1993). In an open-label prospective, phase 1b trial, Leber congenital amaurosis (LCA) patients (aged 6–38 years) with *RPE65* or *LRAT* mutations were given oral QLT091001 (synthetic 9-*cis*-retinyl acetate) for two years and improvement in GVF (Goldmann visual field) areas and visual acuity was observed (Koenekoop et al., 2014). Antioxidants have been reported to reduce oxidative damage to photoreceptors and preserve retinal function in RP models (Komeima et al., 2006). *N*-acetylcysteine amide (NACA) is a potent thiol antioxidant to protect RPE and photoreceptors from oxidative stress by increasing glutathione peroxidase and scavenging ROS (Schimel et al., 2011). Fort Worth-based Nacuity Pharmaceuticals is launching a Phase I/II clinical trial in Australia for NACA oral therapy aiming to slow vision loss in RP patients. Despite the beneficial effects, these supplements can only play protective roles to a certain extent, but do not constitute a real remedy to RP cure. Since *PRPF*-RPs are monogenic IRDs, recent studies have focused primarily on gene-based therapies to rectify *PRPF* mutation-associated abnormalities, which we discuss as follows:

### Augmentation of Normal *PRPF* Expression Level

Haploinsufficiency is a major underlying cause in human RP patients carrying *PRPF31* mutations and the severity of RP is related to the variant expression level of remaining wild type *PRPF31*, thus, gene augmentation is a suitable therapeutic approach for this condition (Rivolta et al., 2006). Recent work has indicated that RPE cells derived from *PRPF31*-RP patients were characterized by decreased *PRPF31* expression and defects in ciliogenesis, reduced cell barrier and phagocytosis (Buskin et al., 2018). When these cells were treated with adeno-associated virus (AAV)-*PRPF31* to supplement the expression of wild type *PRPF31*, aberrant morphological and functional phenotypes were restored, as indicated by increased cilia length and incidence, elevated Transepithelial Electrical Resistance (TER) level, rescued phagocytosis, decreased stress vacuoles and restored collagen IV expression level and pattern in basal lamina (Brydon et al., 2019). These findings indicate that for patients with *PRPF31*-related RP, AAV-based gene augmentation strategy targeting RPE cells holds therapeutic promise. As a single stranded DNA parvovirus, AAV is currently the most attractive vector for gene therapy with benefits including high gene transfer efficiency, stable long-term expression, non-genome integration, low immunogenicity, transduction of both mitotic and postmitotic cells and no pathogenicity to humans (Oner, 2017; Kimura et al., 2019). Several AAV-based vectors have been employed for clinical trials treating retinal diseases such as RP, Usher syndrome, Stargardt disease etc. (Ong et al., 2019). Recently, great progress has been achieved in this field with the approval of LUXTURNA^TM^ by FDA for the gene therapy of LCA, *RPE65* mutation-associated retinal dystrophy (Darrow, 2019). While no clinical trial has been reported for PRPF-related mutation RP gene therapy, it is fair to expect such therapeutic regimens to occur in the not-too-distant future. A major limitation of AAV vector is a relatively small packaging capacity (∼4.7 kb) (Grieger and Samulski, 2005), which is suitable for the transfer of *PRPF3* (2 kb), *PRPF4* (1.6 kb), *PRPF6* (2.8 kb), *PRPF31* (1.5 kb) but not *PRPF8* (7 kb). Nanoparticles have emerged as a promising technology for gene transfer owing to the possession of all AAV merits plus unlimited DNA size (Yanik et al., 2017), making it an ideal alternative vector when it comes to the transfer of large genes.

*PRPF31*-associated adRP features strikingly incomplete penetrance, whereby individual mutation carriers in the affected family exhibit RP symptomatic or asymptomatic (Vithana et al., 2003). The asymptomatic cases have been closely linked to a higher expression of the wild type *PRPF31* allele, implying a particular transcription regulating machinery to compensate for the mutant allele (McGee et al., 1997; Rivolta et al., 2006). Interestingly, *CNOT3* gene that lies near *PRPF31* locus (19q13.4) has shown a significant inverse relationship with *PRPF31* expression. *CNOT3* knockdown led to an increase in *PRPF31* expression and chromatin immunoprecipitation verified the directly binding of CNOT3 protein to a specific *PRPF31* promoter sequence, indicating that CNOT3 acted as a repressive trans-acting factor to regulate *PRPF31* expression (Venturini et al., 2012). The clinical appearance of RP11 tends to be determined by the coinheritance of a heterozygous *PRPF31* mutation and a homozygous higher expressivity variant of *CNOT3* (Rose et al., 2014).

Upstream to the transcription start site (TSS) of *PRPF31* gene, a cluster of MSR1 elements was identified that acts as a *cis* regulatory element to modulate *PRPF31* promoter activity. MSR1 element copy number variation (CNV) regulates the *PRPF31* gene expression, thereby controlling the phenotypic incomplete penetrance in *PRPF31*-associated adRP (Rose et al., 2016). A 3-copy repeat allele dramatically quenched gene expression *in vitro*. *PRPF31* mutation carriers bearing 4-copy repeat MSR1 allele displayed a strong *PRPF31* expression and were asymptomatic. The determination of *PRPF31* expression and RP penetrance by MSR1 copy number suggests that this element might be a candidate regulator for a novel “natural gene therapy.” By linkage analysis, another two *trans*-acting expression quantitative trait loci (eQTL) were established that were strongly related to *PRPF31* expression variation. One of them was located to 19q13.4 that is close to *PRPF31* gene on the wild type allele (McGee et al., 1997). Another one was mapped to chromosome region14q22.1–23.1 that functions as the RP11-distant regulator (Rio Frio et al., 2008). These findings elicit a novel idea that *PRPF31* expression can be enhanced by modulation of its regulatory factors and/or elements, thereby alleviating RP manifestation.

Emerging evidence indicates that most autosomal dominant disorders are mutationally heterogeneous. In addition to haploinsufficiency, gain-of-function or dominant-negative effects contribute to *PRPF*-RPs as well (Supplementary Table 1). Under such circumstances, gene supplementation therapy alone would not be sufficient to restore RP defects. To eliminate the interference of wild type PRPF functions by the mutant protein, specific nullification of the mutant allele in conjunction with gene supplementation may provide a more effective approach (Diakatou et al., 2019). In severe adRP rat model carrying rhodopsin gene S334ter mutation (*Rho*^*S*334^), selective cleavage of *Rho*^*S*334^ site by CRISPR/Cas9 was successfully performed to disrupt the dominant-negative allele and ablate the toxic effects of the mutation, leading to preserved POS structure, enhanced photoreceptor survival and improved visual acuity (Bakondi et al., 2016). Another option is to suppress both the mutant and wild type alleles of a target gene and to supply a replacement gene concurrently, which codes for wild protein but is resistant to suppression due to sequence changes at degenerate sites. In a transgenic mouse model bearing human *RHO* Pro347Ser mutation, AAVshQ1-mediated suppression of human *RHO* in combination with replacement with the endogenous mouse gene significantly delayed the retinal degeneration, demonstrated by photoreceptor preservation and improved ERG (Chadderton et al., 2009). The suppression-and-replacement strategy helps to overcome the mutational heterogeneity present in *PRPF*-adRPs and provides an alternative regiment for prospective clinical practice.

### Gene Editing for Correction of *PRPF* Mutations

A significant downside of gene supplementation therapy for retinal degenerative diseases is the limited effective period in which the vision gains decline over time (Bainbridge et al., 2015; DiCarlo et al., 2018). In this respect, direct correction of the original mutations by gene editing will be more advantageous. Several sequence specific endonuclease systems have been developed for HDR-based gene editing, including Meganucleases, zinc finger nucleases (ZFN), transcription activator-like effector nucleases (TALEN) and clustered regularly interspaced short palindromic repeats (CRISPR/Cas9) (Peng et al., 2017; Yanik et al., 2017).

As mentioned above, we used CRISPR/Cas9 to correct *PRPF31* c.1115_1125del11 mutation in RP11-iPSCs and observed morphology and function restorations in the corrected iPSC-derived photoreceptors and RPE, showing rescued ciliogenesis, phagocytosis and apical-basal polarity, which provided proof of concept for potential therapeutic strategies (Buskin et al., 2018). These *in vitro* gene editing results also point to a promising prospect of *in vivo* correction of *PRPF31* mutations using the CRISPR/Cas9 system. Some work has recently been performed on other IRDs. Autosomal recessive mutation of *RPE65* is the main cause of LCA that leads to childhood-onset blindness. With subretinal injection of CRISPR/Cas9 AAV vector into rd12 LCA mice model, >1% mutation homology-directed repair was achieved and photoreceptor light response was partly rescued (Jo et al., 2019). To restore vision loss in another Leber congenital amaurosis type 10 (LCA10) associated with *CEP290* gene mutations, Maeder et al. (2019) developed a CRISPR/Cas9 therapeutic that efficiently corrected the mutant alleles in humanized mice and primate cells *in vivo*. Recently, this therapeutic approach was approved by FDA for the first phase I/II clinical trial in LCA10 patients, which set the milestone for the treatment of IRDs using the CRISPR/Cas9 tool and kick start the era of genome surgery. Although no *in vivo* CRISPR/Cas9 studies on *PRPF* mutation related RPs have been documented at this time, it is conceivable that such therapeutics are underway. An interesting phenomenon is that most *PRPF8* mutations identified cluster in a highly conserved region within the last exon, suggesting that a possible common CRISPR system might be applicable for the correction of all *PRPF8* mutations (Mordes et al., 2006).

Although significant advances have been made, challenges must be overcome to enable therapeutic use of CRISPR-Cas9 *in vivo*, including vehicle delivery, targeting efficiency, error-prone non-homology end joining (NHEJ), off-targeting etc. (Burnight et al., 2018). More studies are warranted to improve the efficiency, accuracy, and safety of gene editing therapies.

### Other Therapies Independent of Mutation

Gene therapy holds great promise in the treatment of RP, but in most cases, it can only be directed to a specific mutation or a single gene, making it costly and difficult for therapeutic translation. In this regard, generalized therapies suitable for a broad spectrum of retinal degenerative disorders independent of mutation intervention would be welcome for clinical practice.

Rod photoreceptor is the primary affected photoreceptor cell type in RP development followed by secondary cone degeneration. Photoreceptor transcription factor *Nrl* functions as a cell fate switch favoring rod development against cone (Mears et al., 2001). With *Nrl* knockout in *Pde6b*^*rd*1/rd1^ and *Rho*^–/–^ RP mice models, the RP-sensitive rod photoreceptors were reprogrammed into cone-like cells that are resistant to mutation effects and retinal degeneration was substantially suppressed (Montana et al., 2013). Similar results were achieved in another study with two RP animal models rd10 and FVB/N in which CRISPR/Cas9-mediated Nrl inactivation reprogrammed rods into cone-like photoreceptors (Zhu et al., 2017). Along with the *in vivo* rod-to-cone switch, retinal tissue integrity was preserved, and function was significantly improved. Moreover, the retinal rod and cone degeneration were rescued, the outer nuclear layer thickness was improved and the photopic b-wave value increased. These studies showed the therapeutic potential of *in vivo* cell reprogramming in protection of photoreceptors from RP damage and maintenance of retinal function. In addition, the beneficial effects in various RP animal models implicate the universal application of this novel approach in treating RP in a gene- and mutation-independent manner.

The effects of neuroprotective strategies, as compared to gene- or mutation-specific interventions, are not dependent on individual mutations and give a longer care window. In RP animal models and clinical trials for the treatment of geographic atrophy (GA), Neurotrophic Factor (CNTF) has been shown to be highly protective against photoreceptor degradation (Liang et al., 2001; Tao et al., 2002; Zhang et al., 2011). Intravitreal application of rAAV2/2.hCNTF into the *Rho*^–/–^ RP mice model dramatically promoted the survival rate of cone and preserved visual function. Since *PRPF31* mutation leads to the downregulation of *RHO* expression (Yuan et al., 2005), CNTF supplementation might be worth investigating in the treatment of *PRPF*-RP.

Injection of rod-derived cone viability factor (RdCVF) into P23H rat RP model with Rhodopsin mutation decreased cone loss and substantially preserved their function (Yang et al., 2009). Several other neuroprotective neurotrophic factors have shown beneficial effects in RP models, such as brain-derived neurotrophic factor (BDNF), basic fibroblast growth factor (bFGF), pigment epithelium–derived factor (PEDF), and glial cell–derived growth factor (GDNF) (McGee Sanftner et al., 2001; Scholl et al., 2016). Autologous platelet-rich plasma (APRP) contains numerous growth factors (Lubkowska et al., 2012) and subtenon-injection of APRP has been approved for clinical trial for the treatment of RP.

Cell replacement therapy using hiPSC-derived retinal photoreceptor precursors has attracted more and more endeavor for restoration of visual function in RP (Li et al., 2012). In RP animal models, transplanted photoreceptor precursor cells were able to integrate into host retina, re-establish outer nuclear layer and restore visual function (MacLaren et al., 2006; Singh et al., 2013). Phase I/II clinical trials of stem cell based RPE and fetal RPCs have been approved by FDA (Uy et al., 2013; Singh et al., 2020), encouraging great improvement in stem cell therapeutics for the treatment of RP.

## Conclusion

RP-associated PRPF proteins (PRPF3, 4, 6, 8, 31 and SNRNP200) are essential components of U4/U6 and U5 snRNPs, which combine to form tri-snRNPs. Through protein-protein and protein-snRNA interactions, PRPFs play critical roles in the assembly and stabilization of spliceosome, which is essential for RNA splicing. In adRP families, a variety of *PRPF* mutations have been identified, leading to impaired spliceosome assembly, and compromised splicing efficiency. Compared to human RP11-iPSCs and fibroblasts, RP11-RPE and retinal organoids show the most significant enrichment of differentially spliced genes in GO biological processes of pre-mRNA and alternative mRNA splicing, which is also corroborated by the differential exon usage analyses of *Prpf31*^±^ mouse retinae and RPE. The preferential tissue disruption of alternative splicing programs leads to a feed-back loop that exacerbates the splicing deficiencies in retina. In line with the defective splicing machinery, a wide spectrum of mis-splicing and dysregulated genes has been identified in *PRPF*-RP retinal cells, including a variety of retina-specific genes involved in photoreceptor development and functions such as phototransduction, ciliogenesis, retinol metabolism and some involved in generic functions including UPR, cell adhesion and migration, apoptosis etc.

Apart from the essential role of PRPFs in pre-mRNA processing, recent studies have implicated their possible functions beyond RNA splicing. PRPF proteins were found localized to apical inner segment, basal body complex and apical connecting cilium of photoreceptor cells and *PRPF31* mutation have been associated with defective ciliogenesis in RPE and retinal organoids, suggesting that PRPFs may function as important cilia component proteins to take part in retinal development and structural maintenance. Multiple aspects of retinal biological processes are under the regulation of circadian rhythm. Primary RPE cells from *Prpf* mutant mice displayed impaired diurnal rhythmicity of phagocytosis and *Prpf31* mutation led to circadian gene mis-splicing in retinal organoids, indicating a role for PRPFs in retinal circadian regulation. The retina is constantly exposed to exogenous radiation and endogenous ROS; therefore, an intact DNA damage repair system is essential for maintaining genome stability in retinal cells. Various aspects of DNA damage response have been reported to correlate with PRPF functions, including DNA damage recognition, prevention of the R-loop formation and DNA damage repair. Downregulation of *PRPFs* induces defective DNA damage repair and accumulation of R-loop in various cell types including RPE.

The PRPF functions beyond RNA splicing provide novel angles to investigate the pathogenesis of *PRPF*-RP. A thorough understanding of PRPF functions in the retina would help solve the riddle of *PRPF*-RP ontology and inspire the establishment of novel therapeutic strategies. Gene supplementation and CRISPR/Cas9-based gene correction have been tested in *PRPF*-RP patient-derived retinal cells with excellent outcomes, suggesting that these therapies are viable approaches for the treatment of *PRPF*-RPs. A plethora of progress has been obtained in experimental studies or clinical trials on RP regiments, such as neuroprotective factors, *in vivo* cell reprogramming, cell replacement therapy, antioxidants etc. The combination of these mutation-independent methods with mutation-specific gene approaches may provide the platform for the clinical trials that would potentially benefit *PRPF*-RP patients.

## Author Contributions

CY: conception of the work, drafting the work, and provide approval for publication of the content. MG, RA, JC, JA-A, SN-G, CJ, RA, and LA: revising the work and provide approval for publication of the content. SM-J and ML: conception of the work, drafting the work, fund raising, revising the work, and provide approval for publication of the content. All authors: contributed to the article and approved the submitted version.

## Conflict of Interest

The authors declare that the research was conducted in the absence of any commercial or financial relationships that could be construed as a potential conflict of interest.

## Publisher’s Note

All claims expressed in this article are solely those of the authors and do not necessarily represent those of their affiliated organizations, or those of the publisher, the editors and the reviewers. Any product that may be evaluated in this article, or claim that may be made by its manufacturer, is not guaranteed or endorsed by the publisher.

## Footnotes

1
https://sph.uth.edu/retnet/home.htm

2
https://my.qiagendigitalinsights.com/bbp/view/hgmd/pro/start.php

## Supplementary Material

Supplementary Table 1 PRPFs implicated in RP.

Supplementary Table 2 Mis-spliced genes affected by *PRPF31* mutations.

Supplementary Table 3 Pros and cons of different disease models.
