# Supplementary material for: Pre-mRNA Processing Factors and Retinitis Pigmentosa: RNA Splicing and Beyond
Source: Front Cell Dev Biol. 2021 Jul 28;9:700276. doi: 10.3389/fcell.2021.700276 (PMC8355544; doi:10.3389/fcell.2021.700276)
Supplement: Supplementary Table 1 — PRPFs implicated in RP. [file Table_1.DOCX]

**Table S1**. **PRPFs implicated in RP.**

| **Gene name (phenotype)** | **Locus** | **Function** | **OMIM number** | **% of all adRP cases** | **Number of mutations identified** | **Mutation type** | **References** |
| --- | --- | --- | --- | --- | --- | --- | --- |
| *PRPF3* (RP18) | [1q21.2](https://www.omim.org/geneMap/1/970?start=-3&limit=10&highlight=970) | U4/U6 snRNP specific protein, binding of the U4/U6 duplex, maintenance of tri-snRNP stability | [601414](https://www.omim.org/entry/601414) | 1.5% | 10 | dominant-negative or gain-of-function | (Chakarova et al., 2002;Amsterdam et al., 2004;Comitato et al., 2007;Gamundi et al., 2008;Graziotto et al., 2008;Graziotto et al., 2011;Zhong et al., 2016) |
| *PRPF4* (RP70) | [9q32](https://www.omim.org/geneMap/9/395?start=-3&limit=10&highlight=395) | U4/U6 snRNP specific protein, maintenance of tri-snRNP stability | 615922 | rare | 5 | loss-of-function, dominant-negative | (Benaglio et al., 2014;Chen et al., 2014;Linder et al., 2014) |
| *PRPF6* (RP60) | 20q13.33 | U5 snRNP-specific protein, interaction with PRPF31, bridging U4/U6 and U5 snRNP | [613983](https://www.omim.org/entry/613983) | rare | 18 | loss-of-function or dominant-negative? | (Schaffert et al., 2004;Tanackovic et al., 2011) |
| *PRPF8* (RP13) | 17p13.3 | U5 snRNP-specific protein, regulation of BRR2 activity, pre-mRNA splicing | [600059](https://www.omim.org/entry/600059) | 2.6% | 64 | dominant-negative or gain-of-function | (Tarttelin et al., 1996;McKie et al., 2001;Gamundi et al., 2008;Keightley et al., 2013;Farkas et al., 2014) |
| *PRPF31* (RP11) | 19q13.42 | U4/U6 snRNP specific protein, interaction with PRPF6, formation of the tri-snRNP | [600138](https://www.omim.org/entry/600138) | 8.9% | 229 | loss-of-function, dominant-negative | (Al-Maghtheh et al., 1996;McGee et al., 1997;Vithana et al., 2001;Vithana et al., 2003;Yuan et al., 2005;Abu-Safieh et al., 2006;Sullivan et al., 2006;Valdes-Sanchez et al., 2019) |
| *SNRNP200* (RP33) | 2q11.2 | U5 snRNP-specific protein, RNA helicase that catalyzes unwinding of U4/U6 RNA duplices, activation of the spliceosome | 610359 | 1.6% | 45 | dominant-negative | (Bowne et al., 2013;Ledoux and Guthrie, 2016;Ruzickova and Stanek, 2017;Diakatou et al., 2019;Gerth-Kahlert et al., 2019;Zhang et al., 2020) |
| *RP9* (RP9) | 7p14.3 | Interaction with PRPF3, pre-mRNA splicing | 607331 | rare | 6 | ? | (Kim et al., 1995;Keen et al., 2002;Maita et al., 2004;Lv et al., 2017) |

Abu-Safieh, L., Vithana, E.N., Mantel, I., Holder, G.E., Pelosini, L., Bird, A.C., and Bhattacharya, S.S. (2006). A large deletion in the adRP gene PRPF31: evidence that haploinsufficiency is the cause of disease. *Mol Vis* 12**,** 384-388.

Al-Maghtheh, M., Vithana, E., Tarttelin, E., Jay, M., Evans, K., Moore, T., Bhattacharya, S., and Inglehearn, C.F. (1996). Evidence for a major retinitis pigmentosa locus on 19q13.4 (RP11) and association with a unique bimodal expressivity phenotype. *Am J Hum Genet* 59**,** 864-871.

Amsterdam, A., Nissen, R.M., Sun, Z., Swindell, E.C., Farrington, S., and Hopkins, N. (2004). Identification of 315 genes essential for early zebrafish development. *Proc Natl Acad Sci U S A* 101**,** 12792-12797.

Benaglio, P., San Jose, P.F., Avila-Fernandez, A., Ascari, G., Harper, S., Manes, G., Ayuso, C., Hamel, C., Berson, E.L., and Rivolta, C. (2014). Mutational screening of splicing factor genes in cases with autosomal dominant retinitis pigmentosa. *Mol Vis* 20**,** 843-851.

Bowne, S.J., Sullivan, L.S., Avery, C.E., Sasser, E.M., Roorda, A., Duncan, J.L., Wheaton, D.H., Birch, D.G., Branham, K.E., Heckenlively, J.R., Sieving, P.A., and Daiger, S.P. (2013). Mutations in the small nuclear riboprotein 200 kDa gene (SNRNP200) cause 1.6% of autosomal dominant retinitis pigmentosa. *Mol Vis* 19**,** 2407-2417.

Chakarova, C.F., Hims, M.M., Bolz, H., Abu-Safieh, L., Patel, R.J., Papaioannou, M.G., Inglehearn, C.F., Keen, T.J., Willis, C., Moore, A.T., Rosenberg, T., Webster, A.R., Bird, A.C., Gal, A., Hunt, D., Vithana, E.N., and Bhattacharya, S.S. (2002). Mutations in HPRP3, a third member of pre-mRNA splicing factor genes, implicated in autosomal dominant retinitis pigmentosa. *Hum Mol Genet* 11**,** 87-92.

Chen, X., Liu, Y., Sheng, X., Tam, P.O., Zhao, K., Chen, X., Rong, W., Liu, Y., Liu, X., Pan, X., Chen, L.J., Zhao, Q., Vollrath, D., Pang, C.P., and Zhao, C. (2014). PRPF4 mutations cause autosomal dominant retinitis pigmentosa. *Hum Mol Genet* 23**,** 2926-2939.

Comitato, A., Spampanato, C., Chakarova, C., Sanges, D., Bhattacharya, S.S., and Marigo, V. (2007). Mutations in splicing factor PRPF3, causing retinal degeneration, form detrimental aggregates in photoreceptor cells. *Hum Mol Genet* 16**,** 1699-1707.

Diakatou, M., Manes, G., Bocquet, B., Meunier, I., and Kalatzis, V. (2019). Genome Editing as a Treatment for the Most Prevalent Causative Genes of Autosomal Dominant Retinitis Pigmentosa. *Int J Mol Sci* 20.

Farkas, M.H., Lew, D.S., Sousa, M.E., Bujakowska, K., Chatagnon, J., Bhattacharya, S.S., Pierce, E.A., and Nandrot, E.F. (2014). Mutations in pre-mRNA processing factors 3, 8, and 31 cause dysfunction of the retinal pigment epithelium. *Am J Pathol* 184**,** 2641-2652.

Gamundi, M.J., Hernan, I., Muntanyola, M., Maseras, M., Lopez-Romero, P., Alvarez, R., Dopazo, A., Borrego, S., and Carballo, M. (2008). Transcriptional expression of cis-acting and trans-acting splicing mutations cause autosomal dominant retinitis pigmentosa. *Hum Mutat* 29**,** 869-878.

Gerth-Kahlert, C., Koller, S., Hanson, J.V.M., Baehr, L., Tiwari, A., Kivrak-Pfiffner, F., Bahr, A., and Berger, W. (2019). Genotype-Phenotype Analysis of a Novel Recessive and a Recurrent Dominant SNRNP200 Variant Causing Retinitis Pigmentosa. *Invest Ophthalmol Vis Sci* 60**,** 2822-2835.

Graziotto, J.J., Farkas, M.H., Bujakowska, K., Deramaudt, B.M., Zhang, Q., Nandrot, E.F., Inglehearn, C.F., Bhattacharya, S.S., and Pierce, E.A. (2011). Three gene-targeted mouse models of RNA splicing factor RP show late-onset RPE and retinal degeneration. *Invest Ophthalmol Vis Sci* 52**,** 190-198.

Graziotto, J.J., Inglehearn, C.F., Pack, M.A., and Pierce, E.A. (2008). Decreased levels of the RNA splicing factor Prpf3 in mice and zebrafish do not cause photoreceptor degeneration. *Invest Ophthalmol Vis Sci* 49**,** 3830-3838.

Keen, T.J., Hims, M.M., Mckie, A.B., Moore, A.T., Doran, R.M., Mackey, D.A., Mansfield, D.C., Mueller, R.F., Bhattacharya, S.S., Bird, A.C., Markham, A.F., and Inglehearn, C.F. (2002). Mutations in a protein target of the Pim-1 kinase associated with the RP9 form of autosomal dominant retinitis pigmentosa. *Eur J Hum Genet* 10**,** 245-249.

Keightley, M.C., Crowhurst, M.O., Layton, J.E., Beilharz, T., Markmiller, S., Varma, S., Hogan, B.M., De Jong-Curtain, T.A., Heath, J.K., and Lieschke, G.J. (2013). In vivo mutation of pre-mRNA processing factor 8 (Prpf8) affects transcript splicing, cell survival and myeloid differentiation. *FEBS Lett* 587**,** 2150-2157.

Kim, R.Y., Fitzke, F.W., Moore, A.T., Jay, M., Inglehearn, C., Arden, G.B., Bhattacharya, S.S., and Bird, A.C. (1995). Autosomal dominant retinitis pigmentosa mapping to chromosome 7p exhibits variable expression. *Br J Ophthalmol* 79**,** 23-27.

Ledoux, S., and Guthrie, C. (2016). Retinitis Pigmentosa Mutations in Bad Response to Refrigeration 2 (Brr2) Impair ATPase and Helicase Activity. *J Biol Chem* 291**,** 11954-11965.

Linder, B., Hirmer, A., Gal, A., Ruther, K., Bolz, H.J., Winkler, C., Laggerbauer, B., and Fischer, U. (2014). Identification of a PRPF4 loss-of-function variant that abrogates U4/U6.U5 tri-snRNP integration and is associated with retinitis pigmentosa. *PLoS One* 9**,** e111754.

Lv, J.N., Zhou, G.H., Chen, X., Chen, H., Wu, K.C., Xiang, L., Lei, X.L., Zhang, X., Wu, R.H., and Jin, Z.B. (2017). Targeted RP9 ablation and mutagenesis in mouse photoreceptor cells by CRISPR-Cas9. *Sci Rep* 7**,** 43062.

Maita, H., Kitaura, H., Keen, T.J., Inglehearn, C.F., Ariga, H., and Iguchi-Ariga, S.M. (2004). PAP-1, the mutated gene underlying the RP9 form of dominant retinitis pigmentosa, is a splicing factor. *Exp Cell Res* 300**,** 283-296.

Mcgee, T.L., Devoto, M., Ott, J., Berson, E.L., and Dryja, T.P. (1997). Evidence that the penetrance of mutations at the RP11 locus causing dominant retinitis pigmentosa is influenced by a gene linked to the homologous RP11 allele. *Am J Hum Genet* 61**,** 1059-1066.

Mckie, A.B., Mchale, J.C., Keen, T.J., Tarttelin, E.E., Goliath, R., Van Lith-Verhoeven, J.J., Greenberg, J., Ramesar, R.S., Hoyng, C.B., Cremers, F.P., Mackey, D.A., Bhattacharya, S.S., Bird, A.C., Markham, A.F., and Inglehearn, C.F. (2001). Mutations in the pre-mRNA splicing factor gene PRPC8 in autosomal dominant retinitis pigmentosa (RP13). *Hum Mol Genet* 10**,** 1555-1562.

Ruzickova, S., and Stanek, D. (2017). Mutations in spliceosomal proteins and retina degeneration. *RNA Biol* 14**,** 544-552.

Schaffert, N., Hossbach, M., Heintzmann, R., Achsel, T., and Luhrmann, R. (2004). RNAi knockdown of hPrp31 leads to an accumulation of U4/U6 di-snRNPs in Cajal bodies. *EMBO J* 23**,** 3000-3009.

Sullivan, L.S., Bowne, S.J., Seaman, C.R., Blanton, S.H., Lewis, R.A., Heckenlively, J.R., Birch, D.G., Hughbanks-Wheaton, D., and Daiger, S.P. (2006). Genomic rearrangements of the PRPF31 gene account for 2.5% of autosomal dominant retinitis pigmentosa. *Invest Ophthalmol Vis Sci* 47**,** 4579-4588.

Tanackovic, G., Ransijn, A., Ayuso, C., Harper, S., Berson, E.L., and Rivolta, C. (2011). A missense mutation in PRPF6 causes impairment of pre-mRNA splicing and autosomal-dominant retinitis pigmentosa. *Am J Hum Genet* 88**,** 643-649.

Tarttelin, E.E., Plant, C., Weissenbach, J., Bird, A.C., Bhattacharya, S.S., and Inglehearn, C.F. (1996). A new family linked to the RP13 locus for autosomal dominant retinitis pigmentosa on distal 17p. *J Med Genet* 33**,** 518-520.

Valdes-Sanchez, L., Calado, S.M., De La Cerda, B., Aramburu, A., Garcia-Delgado, A.B., Massalini, S., Montero-Sanchez, A., Bhatia, V., Rodriguez-Bocanegra, E., Diez-Lloret, A., Rodriguez-Martinez, D., Chakarova, C., Bhattacharya, S.S., and Diaz-Corrales, F.J. (2019). Retinal pigment epithelium degeneration caused by aggregation of PRPF31 and the role of HSP70 family of proteins. *Mol Med* 26**,** 1.

Vithana, E.N., Abu-Safieh, L., Allen, M.J., Carey, A., Papaioannou, M., Chakarova, C., Al-Maghtheh, M., Ebenezer, N.D., Willis, C., Moore, A.T., Bird, A.C., Hunt, D.M., and Bhattacharya, S.S. (2001). A human homolog of yeast pre-mRNA splicing gene, PRP31, underlies autosomal dominant retinitis pigmentosa on chromosome 19q13.4 (RP11). *Mol Cell* 8**,** 375-381.

Vithana, E.N., Abu-Safieh, L., Pelosini, L., Winchester, E., Hornan, D., Bird, A.C., Hunt, D.M., Bustin, S.A., and Bhattacharya, S.S. (2003). Expression of PRPF31 mRNA in patients with autosomal dominant retinitis pigmentosa: a molecular clue for incomplete penetrance? *Invest Ophthalmol Vis Sci* 44**,** 4204-4209.

Yuan, L., Kawada, M., Havlioglu, N., Tang, H., and Wu, J.Y. (2005). Mutations in PRPF31 inhibit pre-mRNA splicing of rhodopsin gene and cause apoptosis of retinal cells. *J Neurosci* 25**,** 748-757.

Zhang, T., Bai, J., Zhang, X., Zheng, X., Lu, N., Liang, Z., Lin, L., and Chen, Y. (2020). SNRNP200 Mutations Cause Autosomal Dominant Retinitis Pigmentosa. *Front Med (Lausanne)* 7**,** 588991.

Zhong, Z., Yan, M., Sun, W., Wu, Z., Han, L., Zhou, Z., Zheng, F., and Chen, J. (2016). Two novel mutations in PRPF3 causing autosomal dominant retinitis pigmentosa. *Sci Rep* 6**,** 37840.
