# Supplementary material for: Pre-mRNA Processing Factors and Retinitis Pigmentosa: RNA Splicing and Beyond
Source: Front Cell Dev Biol. 2021 Jul 28;9:700276. doi: 10.3389/fcell.2021.700276 (PMC8355544; doi:10.3389/fcell.2021.700276)
Supplement: Supplementary Table 2 — Mis-spliced genes affected by PRPF31 mutations. [file Table_2.DOCX]

**Table S2. Mis-spliced genes affected by *PRPF31* mutations.**

|  |  |  |  |  |
| --- | --- | --- | --- | --- |
| **Function** | **Affected genes** | **Associated disease** | **OMIM** | **Reference** |
| **pre-mRNA splicing** | *PRPF3* | Retinitis Pigmentosa | 601414 | (Buskin et al., 2018;Valdes-Sanchez et al., 2019;Azizzadeh Pormehr et al., 2020) |
|  | *PRPF4* | Retinitis Pigmentosa | 615922 |  |
|  | *PRPF8* | Retinitis Pigmentosa | 600059 |  |
|  | *PRPF19* | Retinitis Pigmentosa | 608330 |  |
|  | *PRPF31* | Retinitis Pigmentosa | 600138 |  |
|  | *SF1* | Multiple Endocrine Neoplasia | [601516](http://omim.org/entry/601516) |  |
|  | *SART1* | Squamous Cell Carcinoma and Renal Cell Carcinoma, Nonpapillary. | [605941](http://omim.org/entry/605941) |  |
|  | *DDX5* | Lung Cancer and Cartilage-Hair Hypoplasia. | [180630](http://omim.org/entry/180630) |  |
|  | *LSM2* | Mixed Connective Tissue Disease and Spinal Muscular Atrophy. | [607282](http://omim.org/entry/607282) |  |
|  | *CPSF1* | Myopia | [606027](http://omim.org/entry/606027) |  |
|  | *U2AF1L4* | Myelophthisic Anemia and Atypical Chronic Myeloid Leukemia. | [601080](http://omim.org/entry/601080) |  |
|  | *DDX39B* | Plasmodium Vivax Malaria and Rheumatoid Arthritis. | [142560](http://omim.org/entry/142560) |  |
|  | *PTPB1* | Human T-Cell Leukemia Virus Type 2 and Patellar Tendinitis. | [600693](http://omim.org/entry/600693) |  |
| **phototransduction** | *RHO* | retinitis pigmentosa; dominant congenital stationary night blindness | 180380 | (Yuan et al., 2005;Mordes et al., 2007;Valdes-Sanchez et al., 2019;Azizzadeh Pormehr et al., 2020) |
|  | *FSCN2* | retinitis pigmentosa; dominant macular dystrophy | 607643 |  |
|  | *RDS* | dominant retinitis pigmentosa; dominant macular dystrophy; digenic RP with ROM1; dominant adult vitelliform macular dystrophy; dominant cone-rod dystrophy; dominant central areolar choroidal dystrophy; recessive LCA. | 179605 |  |
|  | *ROM1* | retinitis pigmentosa | 180721 |  |
|  | *GNAT1* | dominant congenital stationary night blindness, Nougaret type; recessive congenital stationary night blindness | 139330 |  |
|  | *PDE6A* | retinitis pigmentosa | 180071 |  |
|  | *PDE6B* | retinitis pigmentosa; dominant congenital stationary night blindness | 180072 |  |
|  | *ABCA4* | Age-Related Macular Degeneration, Stargardt Disease. | 601691 |  |
| **ciliogenesis** | *ARL6* | retinitis pigmentosa; recessive Bardet-Biedl syndrome | 608845 | (Ivings et al., 2008;Buskin et al., 2018;Valdes-Sanchez et al., 2019;Kaukonen et al., 2021) |
|  | *IFT122* | Retinitis Pigmentosa | 606045 |  |
|  | *IFT27* | recessive Bardet-Biedl syndrome | 615870 |  |
|  | *IFT88* | Retinitis Pigmentosa; Polycystic Kidney Disease | 600595 |  |
|  | *SAG* | Retinitis Pigmentosa; recessive Oguchi disease | 181031 |  |
|  | *RPGR* | Retinitis Pigmentosa, X-Linked, and Sinorespiratory Infections, With or Without Deafness. | 312610 |  |
|  | *RPGRIP1L* | Meckel Syndrome, Type 5 and Joubert Syndrome 7. | 610937 |  |
|  | *SORBS1* | Erythematosquamous Dermatosis and Body Mass Index Quantitative Trait Locus 11. | 605264 |  |
|  | *PTGS1* | Gastric Ulcer; Aspirin Resistance | 176805 |  |
|  | *ODF2* | Spermatogenic Failure; Infertility | 602015 |  |
|  | *IFT80* | Short-Rib Thoracic Dysplasia | 611177 |  |
|  | *PKD2* | Polycystic Kidney Disease 2 With Or Without Polycystic Liver Disease; Autosomal Dominant Polycystic Kidney Disease | 173910 |  |
|  | *RAB15* | Tylosis With Esophageal Cancer; Griscelli Syndrome | 610848 |  |
|  | *DYNC2H1* | Short-Rib Thoracic Dysplasia | 603297 |  |
|  | *BBS1* | Bardet-Biedl Syndrome | 209901 |  |
|  | *BBS4* | Bardet-Biedl Syndrome | 600374 |  |
|  | *BBS5* | Bardet-Biedl Syndrome | 603650 |  |
|  | *BBS7* | Bardet-Biedl Syndrome | 607590 |  |
|  | *BBS9* | Bardet-Biedl Syndrome | 607968 |  |
| **lysosome** | *CLN3* | Juvenile Neuronal Ceroid Lipofuscinosi, non-syndromic Retinitis Pigmentosa. | 607042 | (Buskin et al., 2018) |
|  | *AGA* | Aspartylglucosaminuria; Lysosomal Storage Disease | 613228 |  |
|  | *ANK3* | Mental Retardation; Autosomal Recessive 37; Neuroma | 600465 |  |
|  | *AP5S1* | Spastic Paraplegia | 614824 |  |
|  | *CLN5* | Neuronal Ceroid-Lipofuscinoses | 608102 |  |
|  | *IL4I1* | Striatonigral Degeneration, Infantile and Primary Mediastinal Large B-Cell Lymphoma. | 609742 |  |
|  | *ITM2C* | Cerebral Amyloid Angiopathy, Itm2b-Related, 1 and Cerebral Amyloid Angiopathy, Itm2b-Related, 2. | 609554 |  |
|  | *LAMP2* | Danon Disease and Hypertrophic Cardiomyopathy. | 309060 |  |
|  | *LDLR* | Hypercholesterolemia, Familial, 1 and Homozygous Familial Hypercholesterolemia. | 606945 |  |
|  | *LYN* | Sarcoma and Mastocytosis, Cutaneous. | 165120 |  |
|  | *NAGA* | Kanzaki Disease and Schindler Disease, Type I. | 104170 |  |
|  | *RPTOR* | Tuberous Sclerosis 1 and Tuberous Sclerosis. | 607130 |  |
|  | *SNAP23* | Tetanus and Hemophagocytic Lymphohistiocytosis. | 602534 |  |
|  | *STX3* | Diarrhea 2, With Microvillus Atrophy and Tetanus. | 600876 |  |
|  | *STXBP2* | Hemophagocytic Lymphohistiocytosis | 601717 |  |
|  | *SYT7* | Fetal Akinesia Deformation Sequence 4 and Prostate Cancer. | 604146 |  |
|  | *TIAL1* | Salpingitis Isthmica Nodosa and Ulcerative Blepharitis. | 603413 |  |
|  | *USP4* | Oculopharyngeal Muscular Dystrophy | 603486 |  |
|  | *VPS33B* | Arthrogryposis, Renal Dysfunction, And Cholestasis 1 and Cholestasis. | 608552 |  |
| **transcription regulation** | *CNOT3* | Intellectual Developmental Disorder With Speech Delay, Autism, And Dysmorphic Facies and Precursor T-Cell Acute Lymphoblastic Leukemia. | 604910 | (Buskin et al., 2018) |
| **endoplasmic reticulum** | *RSK* | Coffin-Lowry Syndrome and Tuberous Sclerosis. | 601684 | (Buskin et al., 2018) |
|  | *DNAJC10* | Cutis Laxa, Autosomal Dominant 3 and Cutis Laxa. | 607987 |  |
|  | *FKBP14* | Ehlers-Danlos Syndrome, Kyphoscoliotic Type, 2 and Muscular Dystrophy. | 614505 |  |
|  | *FKBP7* |  | 607062 |  |
|  | *OS9* | Overhydrated Hereditary Stomatocytosis and Bone Cancer. | 609677 |  |
|  | *P4HA2* | Myopia 25, Autosomal Dominant and Myopia. | 600608 |  |
|  | *PDGFC* | Milker's Nodule and Age-Related Macular Degeneration. | 608452 |  |
|  | *PRKCSH* | Polycystic Liver Disease 1 With Or Without Kidney Cysts and Polycystic Liver Disease. | 177060 |  |
|  | *RDH5* | Fundus Albipunctatus and Fundus Dystrophy. | 601617 |  |
|  | *SUMF2* | Multiple Sulfatase Deficiency and Phosphoserine Phosphatase Deficiency. | 607940 |  |
|  | *TOR2A* | Dystonia 1, Torsion, Autosomal Dominant and Blepharospasm. | 608052 |  |
|  | *UGGT1* | Pulmonary Subvalvular Stenosis and Mitochondrial Complex Iv Deficiency, Nuclear Type 1. | 605897 |  |
| **unfolded protein response** | *ACADVL* | Acyl-Coa Dehydrogenase, Very Long-Chain, Deficiency Of and Encephalopathy. | 609575 | (Buskin et al., 2018) |
|  | *ADD1* | Hypertension, Essential and Tracheoesophageal Fistula. | 102680 |  |
|  | *ARFGAP1* | Ceroid Lipofuscinosis, Neuronal, 4B, Autosomal Dominant and Hypotrichosis-Lymphedema-Telangiectasia Syndrome. | 608377 |  |
|  | *CUL7* | Three M Syndrome 1 and Dubowitz Syndrome. | 609577 |  |
|  | *CXXC1* | Zinc Finger Protein 1 and Gait Apraxia. | 609150 |  |
|  | *DCTN1* | Perry Syndrome and Neuronopathy, Distal Hereditary Motor, Type Viib. | 601143 |  |
|  | *SEC31A* | Neurodevelopmental Disorder With Spastic Quadriplegia, Optic Atrophy, Seizures, And Structural Brain Anomalies and Pseudobulbar Palsy. | 610257 |  |
|  | *SEC61A2* |  | 618271 |  |
|  | *SEC62* | Polycystic Liver Disease and Hereditary Lymphedema I. | 602173 |  |
|  | *SYVN1* | Wolfram Syndrome 1 and Wolfram Syndrome. | 608046 |  |
| **serine/threonine-protein kinase that activates AMPK** | *SKT11* | Peutz-Jeghers Syndrome and Testicular Germ Cell Tumor. | 602216 | (Ivings et al., 2008) |
| **cell surface receptor and has been implicated as a regulator of synapse formation, neural plasticity, antimicrobial activity, and iron export.** | *APP* | Cerebral Amyloid Angiopathy, App-Related and Alzheimer Disease. | 104760 | (Tanackovic et al., 2011) |
| **Signaling Pathway** | *FGFR1* | Osteoglophonic Dysplasia and Encephalocraniocutaneous Lipomatosis. | 136350 | (Tanackovic et al., 2011) |
|  | *BMP4* | Microphthalmia, Syndromic 6 and Orofacial Cleft 11. | 112262 |  |
|  | *DDR1* | Meninges Sarcoma and Lymphangioleiomyomatosis. | 600408 |  |
| **apoptosis** | *TRAIL-R2* | Squamous Cell Carcinoma, Head And Neck and Squamous Cell Carcinoma. | 603612 | (Tanackovic et al., 2011) |
|  | *PTPN13* | Streptococcal Meningitis and Colorectal Cancer, Hereditary Nonpolyposis, Type 6. | 600267 |  |
| **post-synaptic membrane** | *UTRN* | Muscular Dystrophy, Becker Type and Muscular Dystrophy. | 128240 | (Tanackovic et al., 2011) |
| **autophagy** | *APG5L* | Spinocerebellar Ataxia, Autosomal Recessive 25 and Stomatitis. | 604261 | (Tanackovic et al., 2011) |
| **DNA repair** | *FANCA* | Fanconi Anemia, Complementation Group A and Pituitary Stalk Interruption Syndrome. | 607139 | (Tanackovic et al., 2011) |

Azizzadeh Pormehr, L., Ahmadian, S., Daftarian, N., Mousavi, S.A., and Shafiezadeh, M. (2020). PRPF31 reduction causes mis-splicing of the phototransduction genes in human organotypic retinal culture. *Eur J Hum Genet* 28**,** 491-498.

Buskin, A., Zhu, L., Chichagova, V., Basu, B., Mozaffari-Jovin, S., Dolan, D., Droop, A., Collin, J., Bronstein, R., Mehrotra, S., Farkas, M., Hilgen, G., White, K., Pan, K.T., Treumann, A., Hallam, D., Bialas, K., Chung, G., Mellough, C., Ding, Y., Krasnogor, N., Przyborski, S., Zwolinski, S., Al-Aama, J., Alharthi, S., Xu, Y., Wheway, G., Szymanska, K., Mckibbin, M., Inglehearn, C.F., Elliott, D.J., Lindsay, S., Ali, R.R., Steel, D.H., Armstrong, L., Sernagor, E., Urlaub, H., Pierce, E., Luhrmann, R., Grellscheid, S.N., Johnson, C.A., and Lako, M. (2018). Disrupted alternative splicing for genes implicated in splicing and ciliogenesis causes PRPF31 retinitis pigmentosa. *Nat Commun* 9**,** 4234.

Ivings, L., Towns, K.V., Matin, M.A., Taylor, C., Ponchel, F., Grainger, R.J., Ramesar, R.S., Mackey, D.A., and Inglehearn, C.F. (2008). Evaluation of splicing efficiency in lymphoblastoid cell lines from patients with splicing-factor retinitis pigmentosa. *Mol Vis* 14**,** 2357-2366.

Kaukonen, M., Pettinen, I.T., Wickstrom, K., Arumilli, M., Donner, J., Juhola, I.J., Holopainen, S., Turunen, J.A., Yoshihara, M., Kere, J., and Lohi, H. (2021). A missense variant in IFT122 associated with a canine model of retinitis pigmentosa. *Hum Genet*.

Mordes, D., Yuan, L., Xu, L., Kawada, M., Molday, R.S., and Wu, J.Y. (2007). Identification of photoreceptor genes affected by PRPF31 mutations associated with autosomal dominant retinitis pigmentosa. *Neurobiol Dis* 26**,** 291-300.

Tanackovic, G., Ransijn, A., Thibault, P., Abou Elela, S., Klinck, R., Berson, E.L., Chabot, B., and Rivolta, C. (2011). PRPF mutations are associated with generalized defects in spliceosome formation and pre-mRNA splicing in patients with retinitis pigmentosa. *Hum Mol Genet* 20**,** 2116-2130.

Valdes-Sanchez, L., Calado, S.M., De La Cerda, B., Aramburu, A., Garcia-Delgado, A.B., Massalini, S., Montero-Sanchez, A., Bhatia, V., Rodriguez-Bocanegra, E., Diez-Lloret, A., Rodriguez-Martinez, D., Chakarova, C., Bhattacharya, S.S., and Diaz-Corrales, F.J. (2019). Retinal pigment epithelium degeneration caused by aggregation of PRPF31 and the role of HSP70 family of proteins. *Mol Med* 26**,** 1.

Yuan, L., Kawada, M., Havlioglu, N., Tang, H., and Wu, J.Y. (2005). Mutations in PRPF31 inhibit pre-mRNA splicing of rhodopsin gene and cause apoptosis of retinal cells. *J Neurosci* 25**,** 748-757.
