# Supplementary material for: Pre-mRNA Processing Factors and Retinitis Pigmentosa: RNA Splicing and Beyond
Source: Front Cell Dev Biol. 2021 Jul 28;9:700276. doi: 10.3389/fcell.2021.700276 (PMC8355544; doi:10.3389/fcell.2021.700276)
Supplement: Supplementary Table 3 — Pros and cons of different disease models. [file Table_3.DOCX]

**Table S3. Pros and cons of different disease models.**

| Model | Pros | Cons |
| --- | --- | --- |
| Yeast | Simplest eukaryote, easy maintenance, complete genome sequence published, genetic modification can be easily achieved, suitable for high-throughput screening, a powerful tool for the study of spliceosome conformation and protein interactions, screening of temperature-sensitive yeast mutants is widely used for identification of pre-mRNA splicing defects. | Non-mammal, far evolutionary distance from human, the characteristics of the yeast cells differ significantly from the human cells and unable to fully mimic disease phenotypes, yeast genome contains much fewer genes than the human genome. |
| Zebrafish | Easy maintenance, yearly round spawning and high fecundity meet demands of experimental specimens, low husbandry costs, high similarity to human genome, genetic modification can be easily achieved, external fertilization and development facilitate observation and manipulation of embryos. | Non-mammal, far evolutionary distance from human, genome annotation is still limited, existence of duplicate genes, limited number of available zebrafish strains, antibodies and cell lines. |
| Mouse | Mammalian model organism, most commonly used model organism, complete mouse genome sequence published, share around 80% of genome with human, plenty mouse disease models available, easy to breed for selective traits, phenotypes of disease models comparable to patients. | Significant physiological and genetic differences with human, high cost of animal maintenance, ethical issue. |
| Human iPSCs | Capability of infinite proliferation and differentiation into all types of cells, no species difference to human, identical genotype as patient donor cells, no risk of immune rejection, avoidance of ethical issue, promising for clinical applications. | High cost of iPSCs culture and differentiation, risk of mutagenesis during reprogramming, risk of tumorgenesis with transplantation. |
